# Transcriptome Sequencing of LYPLA1 Gene Overexpression and Interference

**DOI:** 10.3390/ani16172648

**Published:** 2026-08-24

**Authors:** Yi Yin, Zhanpeng Zhang, Yuan Gou, Huiguo Yang, Kanati Shalike, Zehu Yuan, Wei Sun

**Affiliations:** 1 College of Animal Science and Technology, Yangzhou University, Yangzhou 225009, China; 15995121804@163.com (Y.Y.); 13401726427@163.com (Z.Z.); 2 College of Veterinary Medicine, Yangzhou University, Yangzhou 225009, China; gou1030923@163.com; 3 Institute of Animal Husbandry, Xinjiang Academy of Animal Sciences, Urumqi 830013, China; yanghuiguo3239@163.com (H.Y.); kanati24239@sina.com (K.S.); 4 International Joint Research Laboratory in Universities of Jiangsu Province of China for Domestic Animal Germplasm Resources and Genetic Improvement, Yangzhou University, Yangzhou 225009, China; 5 Joint International Research Laboratory of Agriculture and Agri-Product Safety of Ministry of Education of China, Yangzhou University, Yangzhou 225009, China

**Keywords:** *LYPLA1*, myoblasts, transcriptome sequencing, PI3K-Akt

## Abstract

Previous studies have indicated that changes in *LYPLA1* expression affect the proliferation and differentiation of fetal sheep myoblasts; however, the underlying molecular mechanisms remain unclear. This study examined transcriptional responses to *LYPLA1* overexpression and knockdown in primary myoblasts derived from one Hu sheep fetus and explored the involvement of PI3K-Akt signaling in *LYPLA1*-associated proliferation. RNA sequencing showed that no differentially expressed genes remained significant after false discovery rate correction following *LYPLA1* knockdown, whereas *LYPLA1* overexpression yielded 362 significant genes, including 219 upregulated and 143 downregulated genes. Exploratory enrichment analysis of nominally significant candidate genes implicated extracellular and immune-related functions, steroid hormone biosynthesis, PPAR signaling, cytokine–cytokine receptor interaction, and PI3K-Akt signaling. Pharmacological inhibition with 0.5 μM PI3K/AKT-IN-1 partially attenuated the increases in EdU-positive cells and Cyclin D1 mRNA levels associated with *LYPLA1* overexpression. These findings indicate that *LYPLA1* overexpression is associated with broad transcriptional changes in ovine myoblasts and suggest that PI3K-Akt signaling contributes to the associated proliferative phenotype. However, the underlying mechanism requires further validation.

## Simple Summary

The proliferation and differentiation of sheep muscle cells are important for skeletal muscle development and meat production. In this study, we investigated how changes in *LYPLA1* expression affected gene activity and the proliferation of fetal sheep muscle cells. Increasing *LYPLA1* expression caused broad changes in the activity of genes involved in several biological processes and promoted muscle-cell proliferation, whereas reducing its expression produced much smaller changes. We also found that blocking a cellular signaling pathway involved in cell growth partially reduced the increase in cell proliferation caused by higher *LYPLA1* expression. These findings suggest that *LYPLA1* may contribute to the regulation of sheep muscle-cell growth through changes in gene activity and cell-growth signaling. Further studies are needed to determine the exact molecular mechanisms involved.

## 1. Introduction

Sheep are widely raised because of their strong environmental adaptability and reproductive capacity, making them an economically important livestock species. Growth traits are among the key economic traits that influence sheep production performance and farming profitability; therefore, elucidating the molecular regulatory mechanisms underlying sheep growth and development is of great significance for their genetic improvement and industrial utilization [1]. Skeletal muscle is a critical tissue influencing the growth, development, and meat production performance of livestock, and its formation is finely regulated by processes such as myoblast proliferation, differentiation, and fusion. As key cells in the development and regeneration of skeletal muscle [2], changes in the gene expression patterns of myoblasts can directly influence muscle fiber formation and the muscle development process. Therefore, elucidating the mechanisms of action of key regulatory genes in myoblasts at the transcriptomic level will help to further clarify the molecular basis of muscle growth and development in sheep.

The transcriptome refers to the complete set of RNA molecules expressed by a specific tissue or cell under a given physiological state, primarily comprising mRNA and various non-coding RNAs. Transcriptome sequencing technology can systematically reveal gene expression patterns, regulatory networks, and the biological processes in which they are involved across the entire genome [3]. With the advancement of high-throughput sequencing technologies, RNA sequencing has become an important tool for identifying differentially expressed genes, characterizing transcriptional responses, and generating hypotheses about biological processes and signaling pathways potentially involved in a given phenotype.

The LYPLA1 gene encodes a lipoprotein thioesterase that participates in the depalmitoylation of proteins and may influence cellular biological functions by regulating processes such as membrane protein localization, lipid metabolism, and cell signaling [4]. The PI3K-Akt signaling axis links growth factor sensing, cellular metabolism, cell survival, protein synthesis, and cell cycle regulation. Classic studies have shown that IGF-1 promotes myotube hypertrophy through the mTOR and GSK3 pathways downstream of PI3K/Akt [5], and the Akt/mTOR axis is also a key regulator of the balance between skeletal muscle hypertrophy and atrophy in vivo [6].

Our previous work showed that altering *LYPLA1* expression is associated with changes in the proliferation, differentiation, and myotube formation of fetal sheep myoblasts [7], but the transcriptomic mechanisms underlying these phenotypes remain unresolved. Therefore, the objective of the present study was to define the transcriptional responses to *LYPLA1* overexpression and knockdown and to identify candidate pathways that may mediate *LYPLA1*-associated myoblast proliferation. We hypothesized that modulation of *LYPLA1* expression would alter gene-expression programs related to myoblast growth and that PI3K-Akt signaling would contribute to the proliferative phenotype associated with *LYPLA1* overexpression. To test these hypotheses, we combined FDR-controlled RNA-seq differential-expression and GO/KEGG enrichment analyses with pharmacological inhibition of PI3K-Akt signaling and assessment of EdU incorporation and Cyclin D1 mRNA expression.

## 2. Materials and Methods

### 2.1. Ethical Statement

All animal procedures were approved by the Laboratory Animal Ethics Committee of Yangzhou University (approval no. 202103279; 8 March 2021).

### 2.2. Sample Collection and Isolation and Culture of Myoblasts from Sheep Embryos

The Hu sheep used in this study were provided by Suzhou Taihu Dongshan Goat Industry Development Co., Ltd. (Suzhou, China). One Hu sheep fetus at approximately 85 days’ gestation was selected as the primary cell donor; the fetus’s sex was not recorded. Autoclaved instruments were used to excise the longissimus dorsi muscle under aseptic conditions. The tissue used for cell isolation was kept fresh and immediately subjected to digestion; Tissue samples collected separately for use in other experiments were rapidly frozen in liquid nitrogen and transferred to −80 °C for storage; these frozen tissues were not used for primary cell isolation in this study. Since all sequencing samples in this study were derived from the same donor, each set of three replicates in subsequent analyses represents independent culture replicates rather than culture replicates from distinct animals.

### 2.3. Isolation, Culture, and Identification of Myoblasts from Sheep Fetuses

Primary fetal sheep myoblasts were isolated from fresh longissimus dorsi muscle tissue by digestion with 2 mg/mL type II collagenase for 60 min [8] and were enriched and purified by differential adhesion according to a previously reported method [9]. The cells were cultured at 37 °C in a 5% CO_2_ atmosphere using high-glucose DMEM growth medium supplemented with 10% fetal bovine serum and a penicillin–streptomycin solution. The purified cells were digested with 0.25% trypsin, frozen, and stored in liquid nitrogen. The experiment used cells that had been passaged to the third generation following revival. Induced differentiation was initiated when myoblast confluence reached 80–90%. In vitro myogenic differentiation was performed using an induced differentiation medium (97% high-glucose DMEM, 2% premium horse serum, and penicillin–streptomycin solution), and morphological images of the cells were collected periodically throughout the differentiation process. On day 7 of induced differentiation, myogenic differentiation was assessed via myosin heavy chain (MyHC) immunofluorescence staining. It should be noted that this study did not employ flow cytometry to quantitatively analyze the cellular purity of the cultures; therefore, the potential influence of fibroblasts or other stromal cells on the experimental results cannot be completely ruled out.

### 2.4. Construction of the Overexpression Vector and Synthesis of Small Interfering RNA

The small interfering RNA (siRNA) targeting ovine *LYPLA1*, along with the corresponding negative-control siRNA, was designed and synthesized by Suzhou Jima Biotechnology Co., Ltd. (Suzhou, China). The nucleotide sequences are provided in Table 1. Prior to formal sequencing, the interference effects of siRNA-364, siRNA-208, and siRNA-1947 were compared in myoblasts from the same batch of fetal sheep, and based on the RT-qPCR results, siRNA-364—which exhibited higher interference efficiency—was selected for RNA-seq.

The coding region of the ovine *LYPLA1* gene was amplified by PCR using cDNA derived from sheep tissue as the template, and the primers used for amplification are listed in Table 2. The PCR product was inserted into the pcDNA3.1(+) vector linearized with the restriction enzymes NheI and XhoI, using the ClonExpress II One-Step Cloning Kit according to the manufacturer’s instructions (Vazyme Biotech Co., Ltd., Nanjing, China). The successfully constructed recombinant vector was sent to Nanjing Qingke for verification via Sanger sequencing. After the sequencing results were verified to be correct, the vector was named pcDNA 3.1(+)-*LYPLA1*. The successfully constructed vector was stored at −20 °C for use in subsequent experiments.

### 2.5. Cell Transfection, RNA Extraction, and RT-qPCR

Third-passage cells were selected and seeded into a 6-well plate for culture at approximately 5 × 10^4^ cells per well. Transfection was performed using the jetPRIME transfection reagent (Polyplus, Illkirch, France) according to the manufacturer’s instructions, with the specific reagent amounts listed in Table 3. After passaging, myoblasts were transfected with siRNA targeting the LYPLA1 gene when the cell confluence reached 40–50%, and with a recombinant plasmid overexpressing *LYPLA1* when the cell confluence reached 60–70%. The transfection groups are as follows: the pcDNA 3.1(+) empty vector group versus the pcDNA 3.1(+)-*LYPLA1* group; the pcDNA 3.1(+) empty vector group versus the transfection reagent group; the siRNA-364 group versus the NC group; and the si-NC group versus the transfection reagent group. Each group consisted of three independent culture replicates from the same donor. Cells were evenly distributed among the various treatments, and both types of experiments were conducted in growth medium; differentiation medium was not used.

Cells were cultured for an additional 24–48 h after transfection. When cell confluence reached 95–100%, total RNA was extracted using the TRNzol Universal Total RNA Extraction Kit (Tiangen Biochemical Technology, Beijing, China). A portion of the resulting RNA samples was sent to Beijing Novogene Technology Co., Ltd (Beijing, China) for transcriptome sequencing (samples were provided as TRIzol lysates; the company performed RNA extraction and library preparation for sequencing according to the TRIzol method); another portion was used for technical validation of expression direction via real-time quantitative PCR (RT-qPCR). Samples designated for RT-qPCR validation were purified by chloroform phase separation, isopropanol precipitation, and washing with 75% ethanol. The RNA was subsequently resuspended in RNase-free water, and its concentration and purity were assessed using a microvolume UV spectrophotometer (Suizhen, Hangzhou, China). The purified samples were then stored at −80 °C until further use.

Total RNA was reverse-transcribed into cDNA using the FastKing One-Step Genomic cDNA First-Strand Synthesis Premix Kit (Tiangen Biochemical Technology, Beijing, China). The resulting cDNA was then used as the template for quantitative PCR with ChamQ SYBR qPCR Master Mix (Vazyme Biotech Co., Ltd., Nanjing, China). Three technical replicates were set up for each sample. The total volume of the qPCR reaction mixture was 20 μL, consisting of 10 μL of 2× ChamQ™ SYBR qPCR Master Mix, 0.4 μL each of the forward and reverse primers, 1 μL of cDNA template, and 8.2 μL of nuclease-free water. The amplification program was set according to the reagent manual. After the reaction, melting curve analysis was performed; in this study, data from a single melting peak were selected for analysis. Since the Ct values for *GAPDH* showed minimal variation across treatment groups and culture replicates, it was used for normalization of target gene expression; the relative expression levels of the target genes were calculated using the 2^−ΔΔCt method. All experimental results are presented graphically, with error bars representing standard errors. Gene-specific primers for the target genes were designed using the Primer-BLAST online tool (based on Primer3, version 2.5.0), and detailed primer sequence information is provided in Table 4.

### 2.6. RNA Quality Assessment and Transcriptome Library Construction

After RNA extraction was completed, RNA integrity was assessed using an Agilent 5400 Bioanalyzer, and the RNA concentration and purity of each sample were evaluated. The RNA quality assessment results for each sample are presented in the “RNA Quality Control Results” table in the Supplementary Materials. Samples whose RNA concentration, purity, and integrity all met the sequencing requirements were selected for subsequent transcriptome library construction. The transcriptome library was constructed using the Fast RNA-seq Lib Prep Kit V2 (Cat. No. RK20306) in accordance with the kit instructions. First, Oligo(dT) magnetic beads were used to enrich mRNA with poly(A) tails, followed by random fragmentation of the enriched mRNA under the action of divalent cations. Using the fragmented mRNA as a template, the first strand of cDNA was synthesized via reverse transcription with random primers, and the second strand was synthesized using RNase H and DNA polymerase I to obtain double-stranded cDNA. The purified double-stranded cDNA underwent terminal repair, 3′ A-tailing, and sequencing adapter ligation in sequence. AMPure XP magnetic beads were then used to screen for ligation products with insert lengths of approximately 370–420 bp. The screened products were amplified by PCR and purified again using magnetic beads, ultimately yielding a transcriptome library suitable for high-throughput sequencing.

### 2.7. Library Quality Control and Sequencing

Quality control was performed after library preparation was completed. First, the libraries were uniformly diluted to approximately 1.5 ng/µL. Next, the insert size and fragment distribution of the libraries were analyzed to confirm that they fall within the expected range. Provided that the fragment distribution is normal, a quantitative method was used to accurately measure the effective concentration of the library; libraries with an effective concentration of 1.5 nM or higher were deemed acceptable.

This study included a pcDNA3.1(+) empty vector control group, a pcDNA3.1(+)-*LYPLA1* group, an NC group, and an siRNA-364 group, with three replicates in each group. Library construction, library pooling, and sequencing were performed for all 12 samples within the same batch. Libraries that passed quality control were mixed in equal proportions based on their effective concentration and expected data volume, and subsequently sequenced on the Illumina NovaSeq X Plus high-throughput sequencing platform (Illumina, San Diego, CA, USA). The sequencing strategy was PE150 paired-end sequencing (paired-end 150 bp), in which sequencing was performed from both ends of the library fragments, with a read length of 150 bp per end.

### 2.8. Sequencing Data Quality Control

Raw image data obtained from high-throughput sequencers were first processed using CASAVA software (version 1.8) [10] for base calling and were then converted into raw sequence data in FASTQ format, which included the sequence information of the sequenced fragments and their corresponding quality scores. The raw sequencing data contained a small proportion of reads with adapter contamination, ambiguous bases, or low-quality sequences and were therefore subjected to quality filtering. Reads were removed if they met any of the following criteria: (1) the presence of sequencing adapters; (2) the presence of ambiguous bases; or (3) low-quality bases with Phred scores of Q ≤ 5 accounting for more than 50% of the total read length. After the above filtering, clean reads were obtained, and their Q20, Q30, and GC content were calculated to assess the overall quality and reliability of the data. All subsequent bioinformatics analyses were conducted based on these high-quality clean reads.

### 2.9. Sequence Alignment and Gene Expression Quantification

To improve the alignment accuracy of cross-exon reads, this study used the HISAT2 software (v2.0.5) to align the quality-controlled sequencing data to the sheep reference genome. The reference genome and corresponding annotation files were obtained from the NCBI *Ovis aries* assembly GCF_016772045.2 (ARS-UI_Ramb_v3.0). After sequence alignment was completed, the featureCounts software (v1.5.0) was used to count the number of mapped reads for each gene [11]. Subsequently, raw read counts were normalized for gene length and sequencing depth to calculate the FPKM (fragments per kilobase of transcript per million mapped reads) value for each gene. FPKM was used solely to describe expression patterns and for visualization.

### 2.10. Analysis of Differentially Expressed Genes

Differential expression analysis of the gene-expression count matrix was performed using DESeq2 (v1.20.0) [12]. DESeq2 is based on a negative binomial distribution model, which standardizes the raw gene expression counts and evaluates differences in gene expression levels among different experimental groups by constructing statistical models. To reduce the false positive rate resulting from multiple hypothesis testing, raw *p* values were adjusted using the Benjamini–Hochberg procedure to control the false discovery rate (FDR). Genes with an adjusted *p* value (padj) ≤ 0.05 and an absolute log2 fold change (|log2FoldChange|) ≥ 1.0 were considered significantly differentially expressed genes. Genes with a nominal *p* value ≤ 0.05 and |log2FoldChange| ≥ 1.0 were defined as nominally significant candidate genes. The complete lists of differentially expressed genes and nominally significant candidate genes from the two pairwise comparisons are provided in the Supplementary Materials.

### 2.11. Functional Enrichment Analysis of Differentially Expressed Genes

To explore the biological functions and potential regulatory mechanisms associated with the differentially expressed genes, Gene Ontology (GO) annotation and Kyoto Encyclopedia of Genes and Genomes (KEGG) pathway enrichment analyses were performed using clusterProfiler (v3.8.1) [13]. Enrichment analysis employed statistical methods to assess the degree of enrichment of differentially expressed genes in various GO terms and KEGG pathways. GO terms and KEGG pathways with padj < 0.05 were considered significantly enriched. From the significantly enriched KEGG pathways, we screened for pathways related to cell proliferation as candidate signaling pathways for further study. The complete GO and KEGG enrichment results for all, upregulated, and downregulated differentially expressed genes were provided in Supplementary Materials.

### 2.12. Pharmacological Intervention Experiments on the PI3K-Akt Signaling Pathway

To screen for the optimal working concentration of PI3K-Akt pathway inhibitors, myoblasts were seeded at a density of 3.5 × 10^4^ cells per well in a 12-well plate, and 1 mL of growth medium was added to each well. Once the cell density reached 40–50%, the cells were treated with PI3K-AKT-IN-1 inhibitor (MedChemExpress, Monmouth Junction, NJ, USA, Catalog No.: HY-144806) at final concentrations of 0, 0.5, 1, and 2 μM, respectively. The final concentration of dimethyl sulfoxide (DMSO) was 0.2% in all groups; the 0 μM group was treated with an equal volume of DMSO as a solvent control. Three independent culture replicates were set up for each group. When cell confluence reached 95–100%, total RNA was extracted using the TRNzol Universal Total RNA Extraction Kit (Tiangen Biochemical Technology, Beijing, China). RT-qPCR was used to detect the relative expression levels of *PIK3CA* mRNA, a key upstream gene in the PI3K-Akt signaling pathway. A test concentration that significantly reduced *PIK3CA* mRNA expression was selected as the working concentration for subsequent experiments.

To further validate the association between *LYPLA1*, the PI3K-Akt signaling pathway, and cell proliferation, the following groups were established: the pcDNA3.1(+) empty vector + solvent group, a pcDNA3.1(+) empty vector + inhibitor group, a pcDNA3.1(+) − *LYPLA1* + inhibitor group, and a pcDNA3.1(+) − *LYPLA1* + solvent group, with three independent culture replicates in each group. Transfection with the plasmids was performed when the cell density reached 60%. 12 h after transfection, PI3K/AKT-IN-1 was added directly to the culture medium of the inhibitor-treated groups, while an equal volume of DMSO was added to the corresponding solvent control groups to ensure that the final DMSO concentration remained the same across all groups. After 48 h of culture following plasmid transfection, cells were harvested and total RNA was extracted; relative expression levels of Cyclin D1 mRNA were detected using RT-qPCR. The RT-qPCR primers were designed using the NCBI Primer-BLAST tool (Table 5). The 5-ethynyl-2′-deoxyuridine (EdU) incorporation experiment was performed according to the instructions of the Cell-Light EdU Apollo In Vitro Kit (Guangzhou Ruibo Biotechnology Co., Ltd., Guangzhou, China), and cell proliferation levels were evaluated by calculating the proportion of EdU-positive cells relative to the total cell count.

## 3. Results

### 3.1. Culture and Identification of Myoblasts from Sheep Fetal Tissue

**Figure 1:**
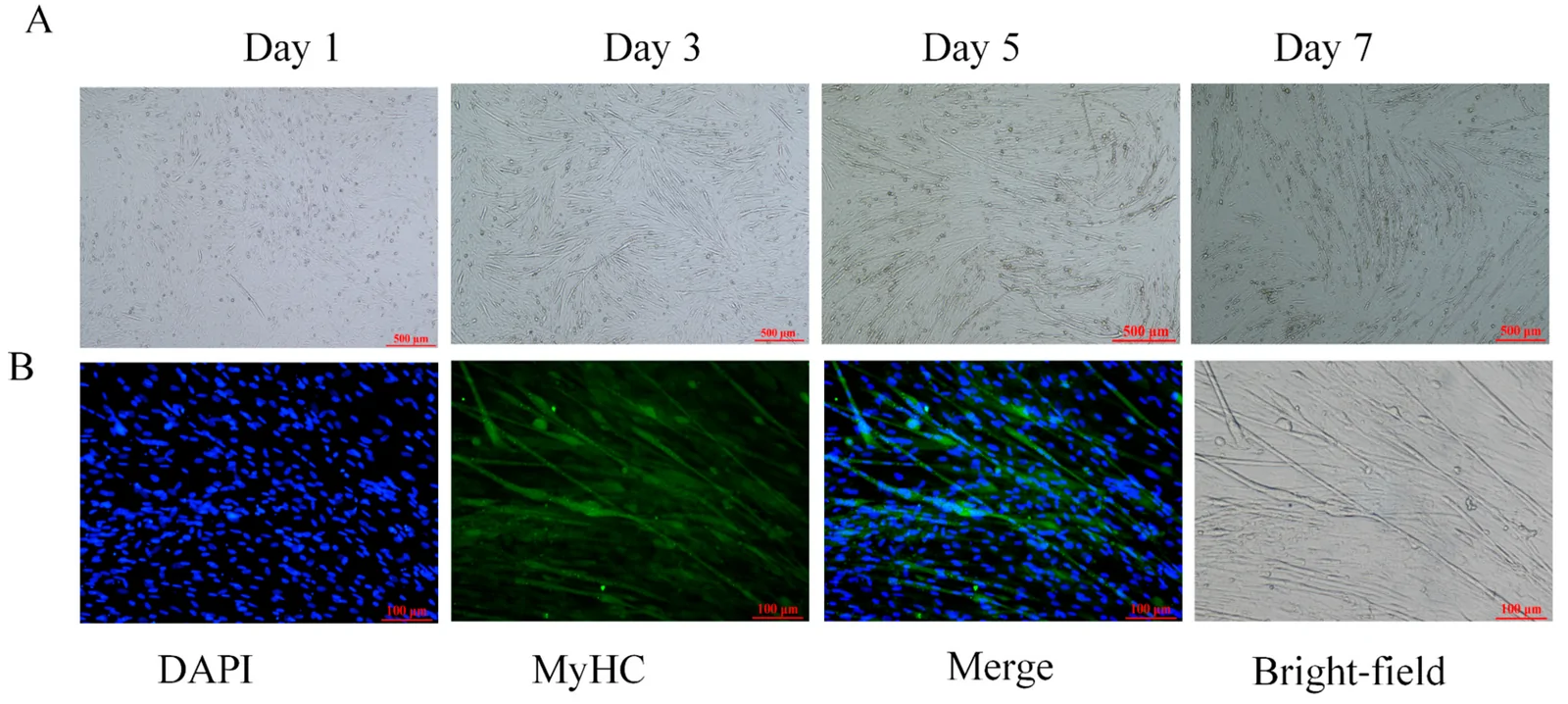
Culture morphology and myogenic differentiation potential of ovine fetal myoblasts. (**A**): Representative bright-field images of ovine fetal myoblasts on days 1, 3, 5, and 7 of induced differentiation; (**B**): Representative images of ovine fetal myoblasts on day 7 of induced differentiation, showing DAPI staining of cell nuclei (blue), immunofluorescence staining of myosin heavy chain (MyHC; green), merged fluorescence, and the corresponding bright-field image.

As shown in Figure 1A, as the duration of induced differentiation increased, the cells gradually elongated and arranged themselves in an oriented pattern, subsequently fusing with one another to form elongated, multinucleated myotubes; by day 7 of induction, a large number of densely packed, well-formed myotube structures could be observed. Immunofluorescence staining results on day 7 of induced differentiation (Figure 1B) showed marked positive expression of myosin heavy chain (MyHC); the MyHC-positive myotubes exhibited intact morphology and were arranged in bundles. These results indicate that the cells isolated in this study support the myogenic identity and differentiation potential of the cultured cells; however, they do not provide a quantitative estimate of myoblast purity.

### 3.2. Validation of LYPLA1 Knockdown and Overexpression Efficiency

**Figure 2:**
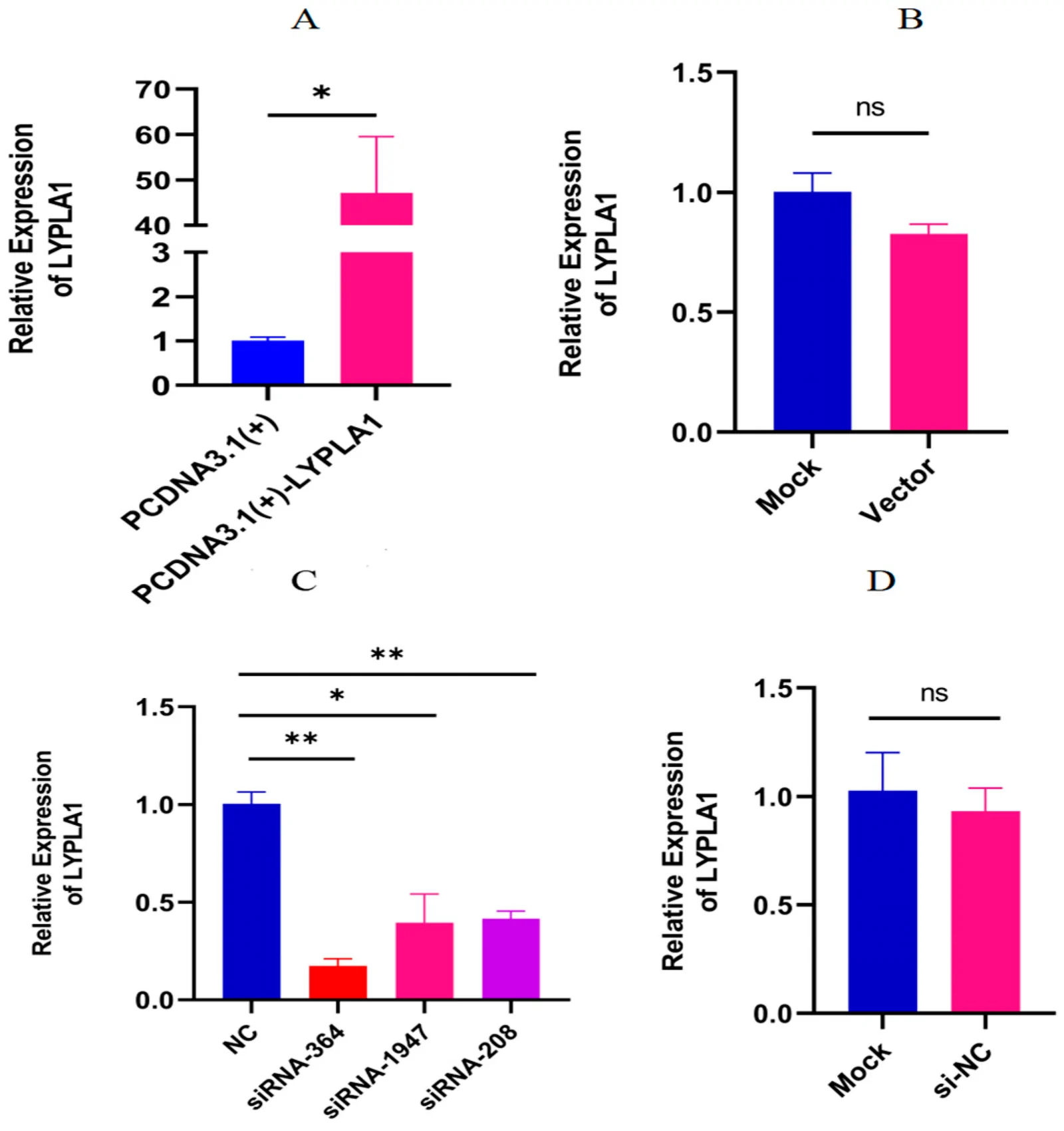
Validation of *LYPLA1* siRNA and OE Efficacy (**A**): Assessment of *LYPLA1* overexpression efficiency; (**B**): Comparison of *LYPLA1* expression levels between the mock group and the empty vector group; (**C**): Assessment of the siRNA interference efficiency of three *LYPLA1*-targeting siRNAs; (**D**): Comparison of *LYPLA1* expression levels between the mock group and the si-NC group. Data are presented as mean ± SEM, n = 3; ns, *p* > 0.05; *, *p* < 0.05; **, *p* < 0.01.

As shown in Figure 2, compared with the empty vector control group, *LYPLA1* mRNA expression levels in the cells were significantly elevated following transfection with the *LYPLA1* overexpression plasmid, indicating that the *LYPLA1* overexpression model was successfully established (Figure 2A). There was no statistically significant difference in *LYPLA1* expression between the Mock group and the empty vector control group (Figure 2B), suggesting that the transfection procedure and the empty vector itself did not significantly affect *LYPLA1* expression. Compared with the negative control (NC) group, all three siRNAs targeting *LYPLA1* significantly reduced *LYPLA1* mRNA expression levels; among them, siRNA-364 exhibited the highest silencing efficiency and was therefore selected for subsequent RNA-seq analysis (Figure 2C). Furthermore, there was no statistically significant difference in *LYPLA1* expression between the Mock group and the si-NC group (Figure 2D), suggesting that the negative control siRNA and the transfection procedure itself did not significantly affect *LYPLA1* expression.

### 3.3. Quality Analysis of Transcriptome Sequencing Data

Quality control and statistical analysis were performed on the raw sequencing data for each sample. The results are shown in Table 6, and more detailed data are presented in the Supplementary Materials. After quality control, the number of clean reads obtained for each sample exceeded 4.5 × 10^7^, meeting the depth requirements for subsequent transcriptomic analysis. The Q20 values for all samples were above 99%, and the Q30 values were above 96%; the GC content ranged from 51% to 56%. These results indicate a high level of sequencing base accuracy and reliable overall data quality.

To evaluate the overall quality and biological reproducibility of the transcriptome sequencing data, this study performed principal component analysis, Pearson correlation analysis, and gene expression distribution analysis on the *LYPLA1* overexpression experiment and the siRNA interference experiment, respectively.

**Figure 3:**
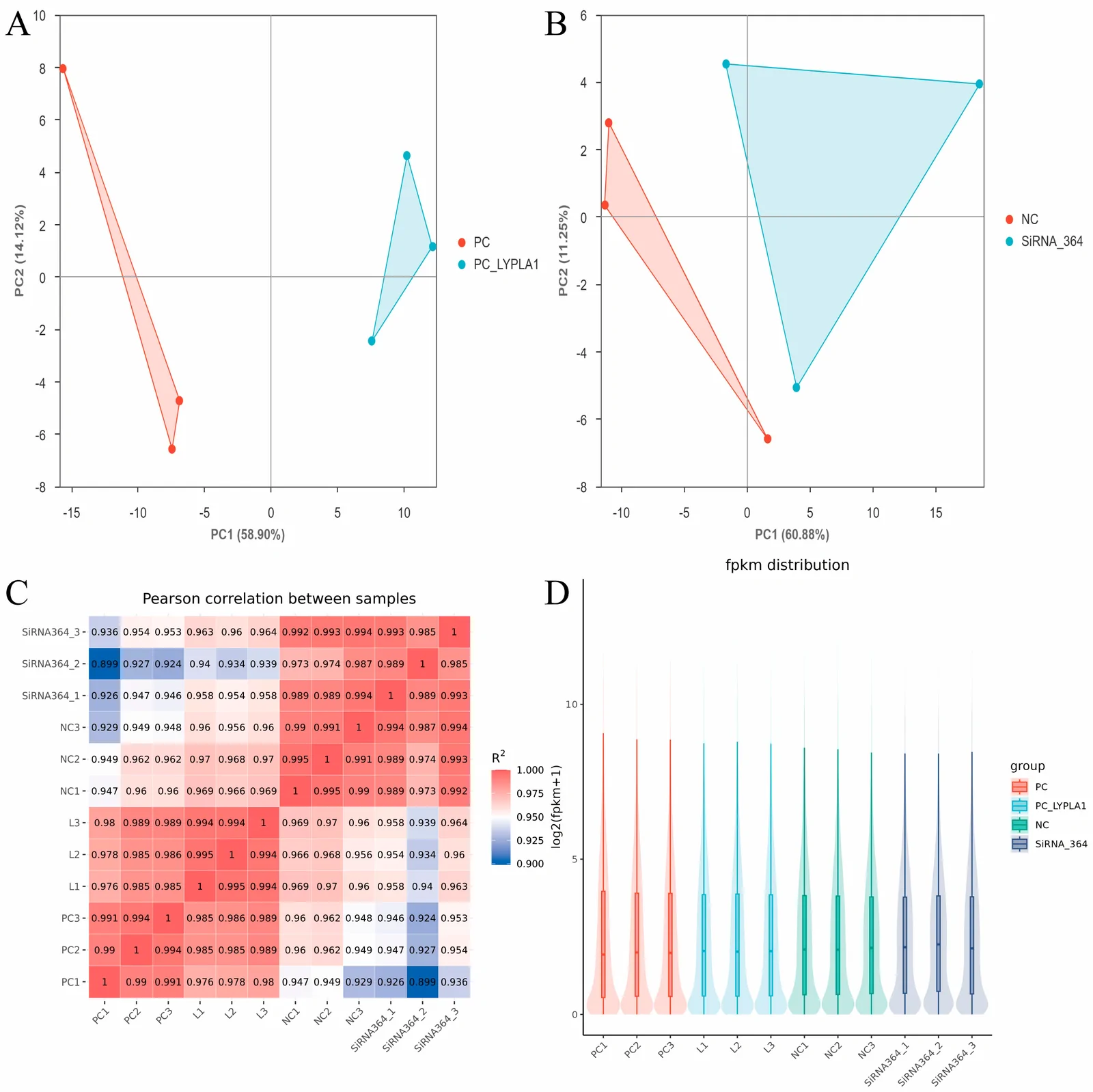
Quality Analysis of Transcriptome Sequencing Data for Each Sample (**A**): Principal component analysis of the *LYPLA1* overexpression group versus the empty vector control group; (**B**): Principal component analysis of the siRNA-364 group versus the negative control group; (**C**): Heatmap of Pearson correlation analysis among samples; (**D**): Violin plot of gene expression levels across samples. Note: PC and PC_*LYPLA1* represent the empty vector control group and the *LYPLA1* overexpression group, respectively, in the *LYPLA1* overexpression experiment; NC and siRNA_364 represent the negative control group and the siRNA-364 treatment group, respectively, in the *LYPLA1* knockdown experiment. PCA is used to describe the overall expression patterns of the samples and does not imply that differences between groups have been verified by inferential statistical tests.

In the *LYPLA1* overexpression experiment, PC1 and PC2 explained 58.90% and 14.12% of the total variance, respectively, and the *LYPLA1* overexpression group and the empty vector control group showed a tendency to separate in the principal component space (Figure 3A). In the *LYPLA1* knockdown experiment, PC1 and PC2 explained 60.88% and 11.25% of the total variance, respectively, and the siRNA-364 group and the negative control group also showed a certain degree of separation (Figure 3B). These results suggest that there may be differences in the overall transcriptional expression profiles among the different treatment groups. It should be noted that PCA is a descriptive analysis; its results are used solely to illustrate overall expression patterns across samples and cannot be used alone to determine whether differences between groups are statistically significant.

Pearson correlation analysis showed that, overall, there was a high degree of correlation among the samples, and culture replicates within the same group exhibited good consistency; however, the correlation was relatively lower for some samples in different treatment groups, suggesting that there may be certain differences in the overall expression patterns between the treatment groups and their corresponding control groups (Figure 3C). Analysis of gene expression distribution showed that the median expression levels and overall distribution ranges of the samples were relatively similar, with no obvious abnormal shifts observed, indicating good comparability among the samples (Figure 3D). In summary, the culture replicates within each group were generally consistent, and the distribution of gene expression levels was relatively stable, supporting the conduct of differential expression analyses in the two experimental systems separately. Given the differences in transfection methods and experimental procedures between the plasmid overexpression and siRNA interference experiments, this study does not provide a biological interpretation of the overall differences between the two experimental systems based on the combined PCA results.

### 3.4. Results of the Screening for Nominally Significant Candidate Genes Between the siRNA-364 Group and the NC Group

To investigate the effects of *LYPLA1* siRNA treatment on cellular gene expression, this study conducted a differential gene expression analysis comparing the siRNA-364 group with the NC group.

**Figure 4:**
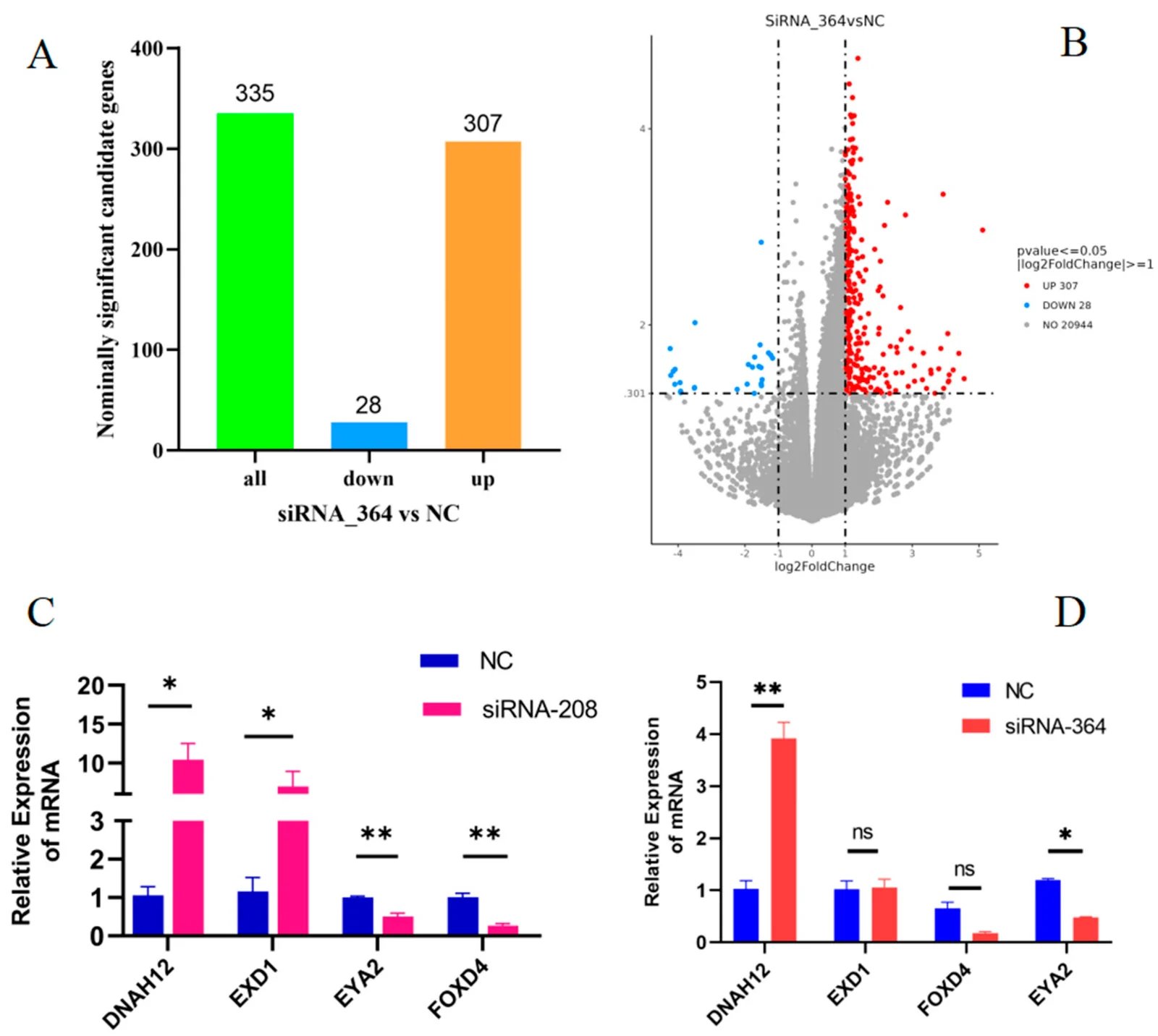
Identification and RT-qPCR validation of nominally significant candidate genes. (**A**): Number of nominally significant candidate genes identified in the siRNA-364 group compared with the negative control (NC) group; (**B**): Volcano plot of nominally significant candidate genes between the siRNA-364 and NC groups. Red dots indicate nominally significantly upregulated genes, blue dots indicate nominally significantly downregulated genes, and gray dots indicate genes without significant differential expression; (**C**): RT-qPCR validation of the nominally significant candidate genes in the siRNA-208 and NC groups; (**D**): RT-qPCR validation of the nominally significant candidate genes in the siRNA-364 and NC groups. Data are presented as the mean ± SEM. ns: *p* > 0.05; *: *p* < 0.05; **: *p* < 0.01; n = 3.

After re-screening using the criteria of padj < 0.05 and |log2FoldChange| ≥ 1, this study did not identify any differentially expressed genes that met these criteria; there were zero up-regulated and zero down-regulated genes. Therefore, this study adopted the more lenient criteria of an original *p*-value < 0.05 and |log2FoldChange| ≥ 1. As shown in Figure 4A, a total of 335 nominally significant candidate genes were identified between the two groups. The complete list of nominally significant candidate genes was provided in Supplementary Materials, including 307 up-regulated genes (such as *DNAH12* and *EXD1*) and 28 down-regulated genes (such as *FOXD4*, *EYA2*, and *GNG13*). The gene with the highest upregulation fold was *DNAH12* (*p* = 1.08 × 10^−3^), and the gene with the highest downregulation fold was *FOXD4* (*p* = 1.74 × 10^−2^). Table 7 and Table 8 list the top 5 up-regulated and down-regulated nominally significant candidate genes, respectively, sorted by log2FoldChange. The volcano plot illustrates the overall distribution of nominally significant candidate genes between the two groups in terms of fold change and significance level (Figure 4B), showing that nominally significant candidate genes are primarily concentrated in the upregulated region, while the number of downregulated genes is relatively small. To further validate the reliability of the transcriptomic results, a subset of nominally significant candidate genes was selected for quantitative RT-qPCR analysis. Following siRNA-364 interference, *DNAH12* was significantly upregulated and *EYA2* was significantly downregulated, while changes in *EXD1* and *FOXD4* were not statistically significant; however, the overall trend of these changes was generally consistent with the RNA-seq results (Figure 4D). Furthermore, to assess the potential impact of sequence-specific off-target effects, this study used another independent siRNA fragment, siRNA-208, targeting *LYPLA1*, to validate selected nominally significant candidate genes via RT-qPCR. The results showed that *DNAH12* and *EXD1* expression were significantly increased, while *EYA2* and *FOXD4* expression were significantly decreased, consistent with the direction of changes observed in the siRNA-364 group via RNA-seq (Figure 4C). The two siRNAs, which target different sites, yielded expression changes with the same direction, further supporting that the changes in these candidate genes are associated with reduced *LYPLA1* expression. Given that the aforementioned genes did not reach FDR significance in the RNA-seq analysis, their potential biological roles still require further validation in a larger number of independent samples.

### 3.5. Exploratory GO and KEGG Enrichment Analysis of Nominally Significant Candidate Genes Following LYPLA1 Knockdown

**Figure 5:**
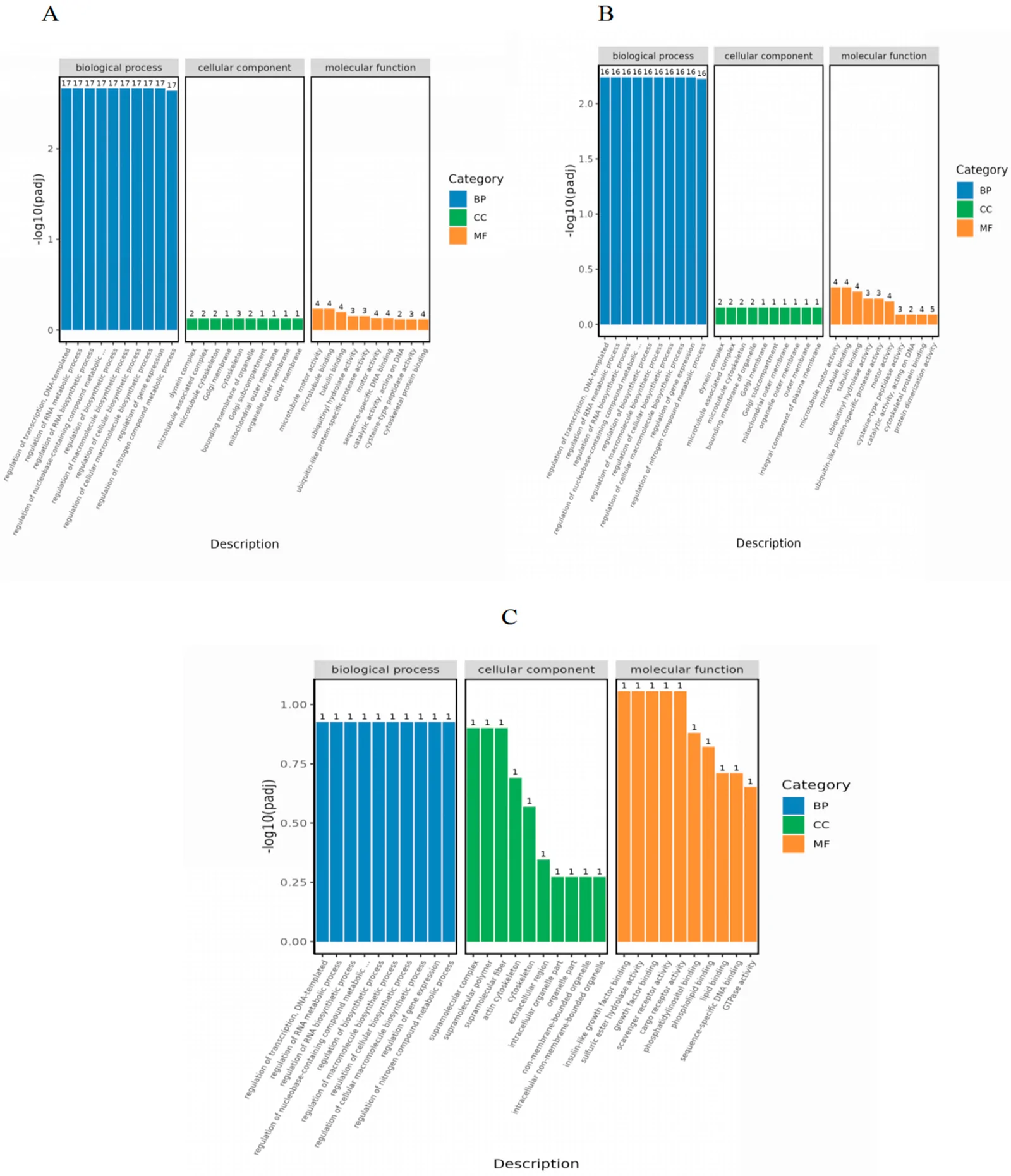

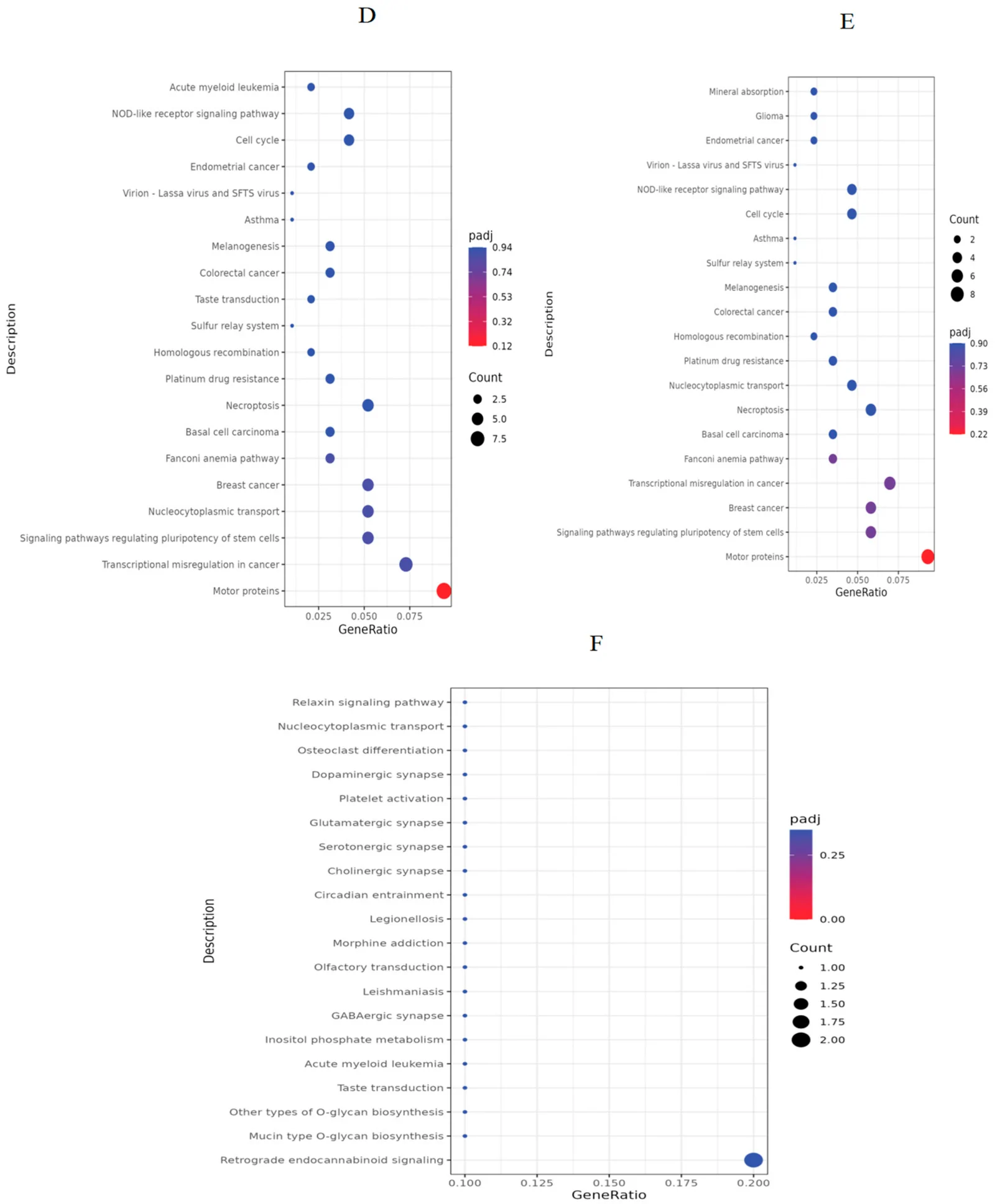
GO and KEGG enrichment analyses of nominally significant candidate differentially expressed genes between the *LYPLA1* siRNA and negative-control groups. (**A**): GO enrichment bar plot of all candidate differentially expressed genes. (**B**): GO enrichment bar plot of genes upregulated in the *LYPLA1* siRNA group. (**C**): GO enrichment bar plot of genes downregulated in the *LYPLA1* siRNA group. (**D**): KEGG pathway enrichment bubble plot of all candidate differentially expressed genes. (**E**): KEGG pathway enrichment bubble plot of genes upregulated in the *LYPLA1* siRNA group. (**F**): KEGG pathway enrichment bubble plot of genes downregulated in the *LYPLA1* siRNA group.

Enrichment analyses were performed using all genes included in the differential expression analysis and successfully annotated in the GO or KEGG database as the background gene set, whereas candidate differentially expressed genes successfully annotated in the corresponding database were used as the query gene set. Among the 335 candidate differentially expressed genes, 59, 39, and 114 genes were annotated to the Biological Process (BP), Cellular Component (CC), and Molecular Function (MF) ontologies of the GO database, respectively. The corresponding effective background gene sets comprised 4154, 2647, and 6844 genes. GO enrichment analysis of all candidate differentially expressed genes identified 287 terms, including 118 BP terms, 61 CC terms, and 108 MF terms (Figure 5A). Among them, 16 BP terms were significantly enriched, whereas no significant CC or MF terms were detected. The significantly enriched BP terms were mainly associated with DNA-templated transcriptional regulation, regulation of RNA metabolism and biosynthesis, gene expression, and metabolic processes. Nine GO terms shared the lowest adjusted *p* value (padj = 0.00216), including regulation of transcription, DNA-templated, regulation of RNA metabolic process, and regulation of RNA biosynthetic process. Each term contained 17 candidate differentially expressed genes, of which 16 were upregulated and one was downregulated. In addition, microtubule-based process (*p* adj = 0.0245) and microtubule-based movement (*p* adj = 0.0479) were significantly enriched and contained five and four upregulated genes, respectively. Separate GO enrichment analyses of the upregulated and downregulated candidate differentially expressed genes identified 278 and 49 GO terms, respectively. Among the upregulated genes, 16 BP terms were significantly enriched, whereas no significant CC or MF terms were detected (Figure 5B). In contrast, none of the GO terms identified for the downregulated genes reached statistical significance (Figure 5C). These results suggest that the transcriptional changes induced by *LYPLA1* silencing may be mainly associated with transcriptional and RNA metabolic regulation, as well as microtubule-related processes.

To further characterize the signaling pathways involving the candidate differentially expressed genes, KEGG pathway enrichment analysis was performed using all genes included in the differential expression analysis and successfully annotated in the KEGG database as the background gene set. Of the 335 candidate differentially expressed genes, 96 were successfully annotated in the KEGG database, including 86 upregulated and 10 downregulated genes, with an effective background gene set of 7839 genes. A total of 170 KEGG pathways were identified for all candidate differentially expressed genes, but none reached statistical significance (padj > 0.05; Figure 5D). Among these pathways, Motor proteins had the lowest padj value (padj = 0.1195) and contained nine candidate differentially expressed genes, including eight upregulated genes and one downregulated gene. Separate KEGG analyses of the upregulated and downregulated genes identified 152 and 46 pathways, respectively. Motor proteins showed the lowest padj value among the upregulated genes (padj = 0.22; Figure 5E), whereas Retrograde endocannabinoid signaling showed the lowest padj value among the downregulated genes (padj = 0.35; Figure 5F). Neither pathway reached statistical significance. These findings indicate that the candidate differentially expressed genes induced by *LYPLA1* silencing were not significantly enriched in any specific KEGG pathway.

### 3.6. Results of the Screening for Differentially Expressed Genes Between the pcDNA3.1(+)-LYPLA1 Group and the pcDNA3.1(+) Group

**Figure 6:**
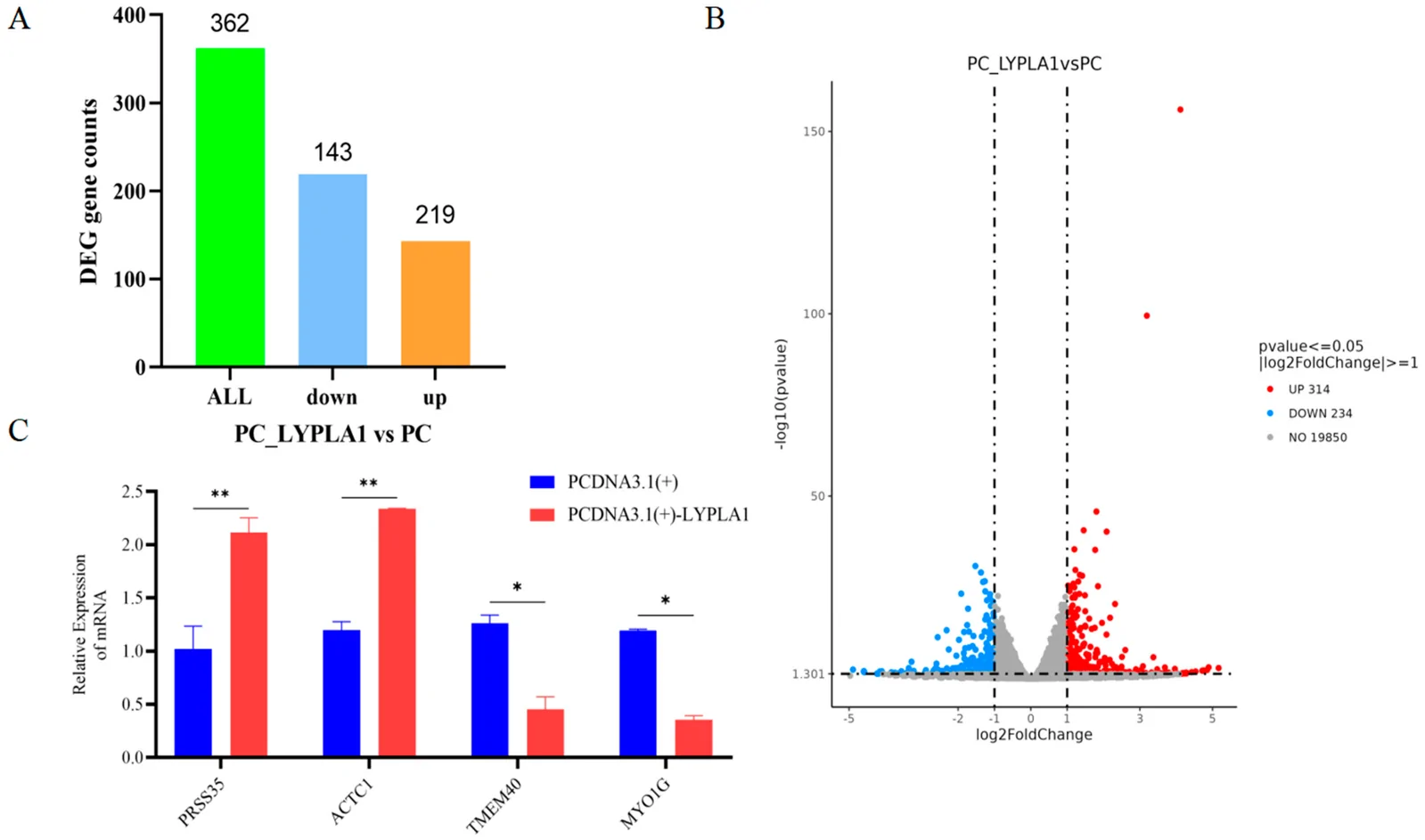
Statistics on Differentially Expressed Genes in the Overexpression Group (**A**): Statistics on the number of differentially expressed genes between PC-*LYPLA1* and PC; (**B**): Volcano plot of nominally significant candidate genes between PC-*LYPLA1* and PC; (**C**): RT-qPCR confirmation of selected genes in the overexpression group; *: *p* < 0.05; **: *p* < 0.01; n = 3.

To investigate the effects of *LYPLA1* overexpression on the transcriptomic profile of sheep myoblasts, differential expression analysis was performed between the pcDNA3.1(+) − *LYPLA1* group and the pcDNA3.1(+) empty vector control group. Complete information on the differentially expressed genes was provided in the Supplementary Materials. Based on the criteria of padj < 0.05 and |log2FoldChange| ≥ 1, this study identified a total of 362 differentially expressed genes, of which 219 were upregulated and 143 were downregulated (Figure 6A). When using a more lenient criterion of *p*-value < 0.05, the number of nominally significant candidate genes was 548 (314 upregulated and 234 downregulated) (Figure 6B). Table 9 and Table 10 list the top 5 upregulated and downregulated differentially expressed genes, respectively, sorted by log2FoldChange. Among the upregulated genes, *LYPLA1*, *GPM6B*, *PRSS35*, *ACTC1*, and others showed relatively pronounced changes in expression (Table 9). Notably, *LYPLA1* was significantly upregulated in the overexpression group (padj = 1.41 × 10^−152^), indicating that *LYPLA1* overexpression was confirmed at the mRNA level; the downregulated genes primarily included *TMEM40*, *MYO1G*, *CSF3*, *CSF2*, and others (Table 10). This study further conducted quantitative validation of the differentially expressed genes, and the results showed that their expression trends were generally consistent with the transcriptome sequencing results (Figure 6C). In summary, *LYPLA1* overexpression significantly alters the gene expression profile of sheep myoblasts, suggesting that *LYPLA1* may be involved in regulating myoblast-related transcriptional networks and providing a basis for subsequent functional enrichment analysis and studies on molecular mechanisms.

### 3.7. GO and KEGG Enrichment Analysis of Candidate Differentially Expressed Genes Between the pcDNA3.1(+)-LYPLA1 and the pcDNA3.1(+) Groups

**Figure 7:**
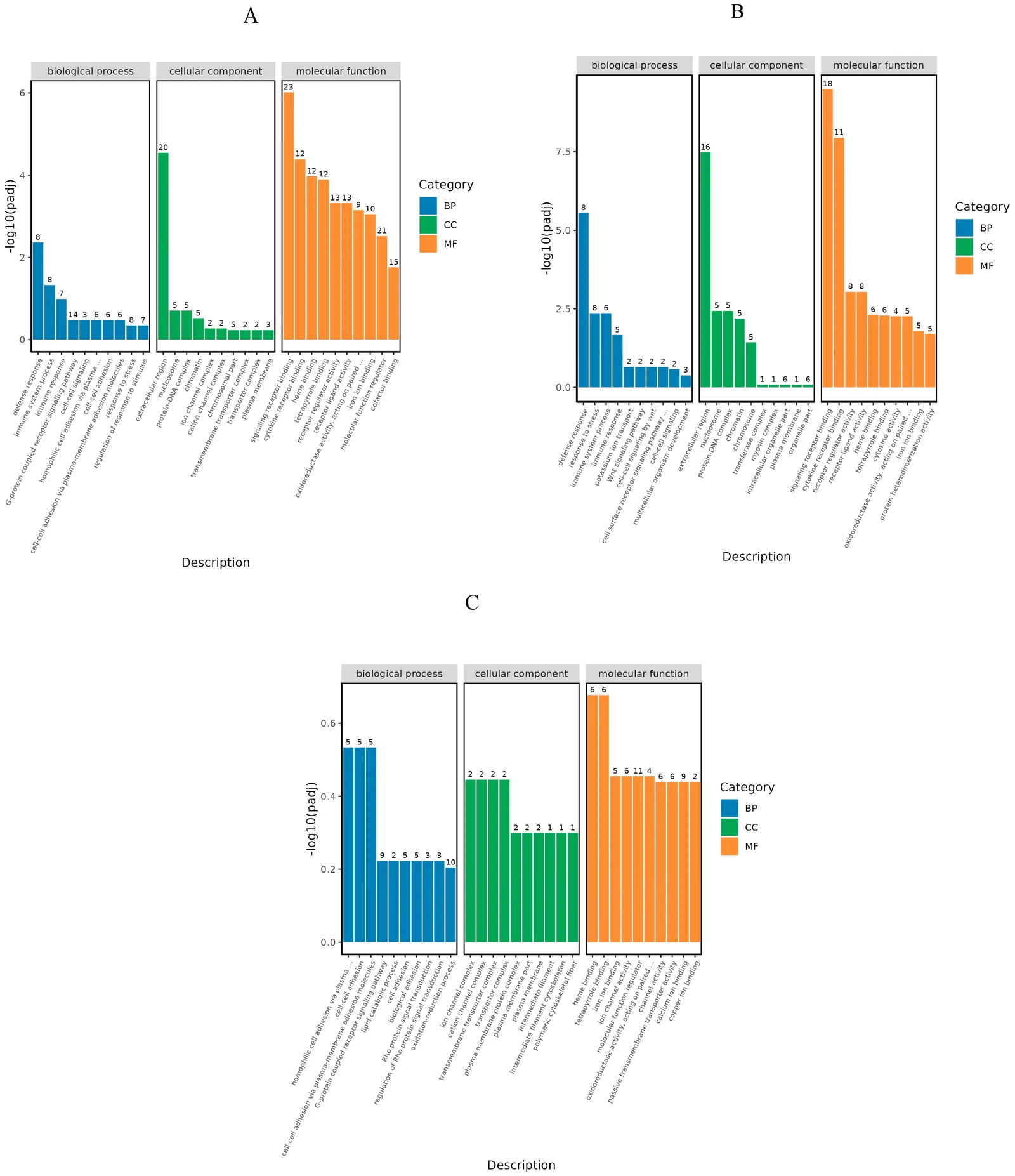

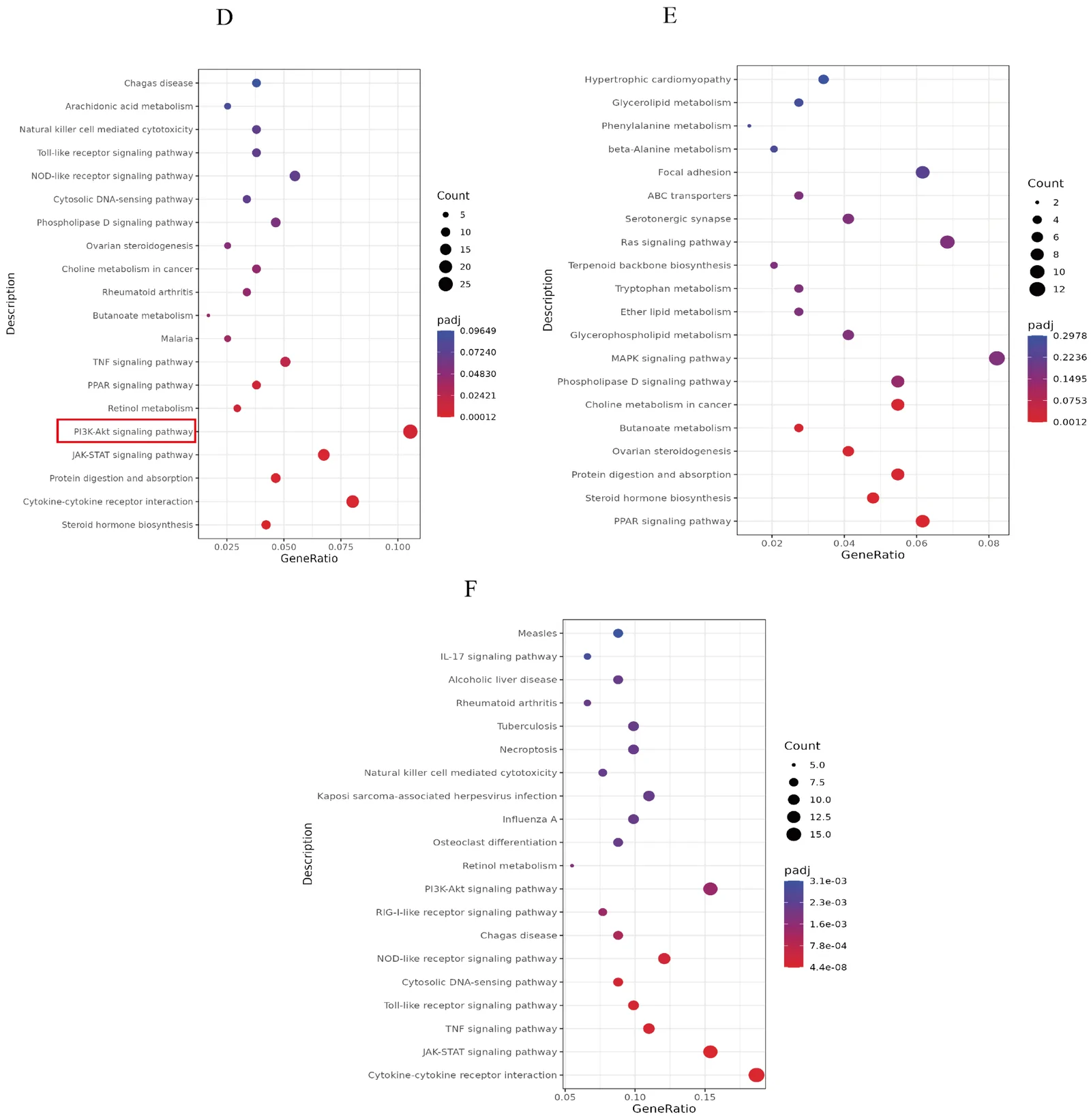
GO and KEGG enrichment results for candidate differentially expressed genes in the *LYPLA1* overexpression group (**A**): Bar chart showing GO functional enrichment for all nominally significant candidate genes in the overexpression group; (**B**): Bar chart showing GO functional enrichment for nominally downregulated candidate genes in the overexpression group; (**C**): Bar chart showing GO functional enrichment for nominally upregulated candidate genes in the overexpression group; (**D**): Bubble plot showing KEGG pathway enrichment for all nominally significant candidate genes in the overexpression group; (**E**): Bubble plot showing KEGG functional enrichment for nominally upregulated candidate genes in the overexpression group; (**F**): Bubble plot showing KEGG functional enrichment for nominally downregulated candidate genes in the overexpression group.

To elucidate the potential biological functions associated with *LYPLA1* overexpression, GO and KEGG enrichment analyses were performed on the candidate differentially expressed genes between the pcDNA3.1(+)-*LYPLA1* (PC-*LYPLA1*) and pcDNA3.1(+) (PC) groups. All genes included in the differential expression analysis and successfully annotated in the corresponding GO or KEGG database were used as the background gene set, whereas candidate differentially expressed genes successfully annotated in the corresponding database were used as the query gene set. Statistical significance was defined as padj < 0.05. Figure 7 presents representative GO terms and KEGG pathways, and the complete results for all candidate differentially expressed genes and the upregulated and downregulated subsets were provided in Supplementary Materials. Among the 548 candidate differentially expressed genes selected based on nominal *p* values, 135, 83, and 216 genes were successfully annotated to the Biological Process (BP), Cellular Component (CC), and Molecular Function (MF) ontologies, respectively. The corresponding effective background gene sets comprised 4035, 2564, and 6655 genes. Because an individual gene may be annotated to more than one GO ontology, these numbers were not additive. GO enrichment analysis of all candidate differentially expressed genes identified 396 terms, including 200 BP, 39 CC, and 157 MF terms (Figure 7A). Fourteen terms were significantly enriched, comprising two BP, one CC, and 11 MF terms. The significant BP terms were defense response (padj = 0.00434) and immune system process (padj = 0.0467), whereas extracellular region was the only significant CC term (padj = 2.85 × 10^−5^). Among the MF terms, signaling receptor binding showed the smallest adjusted *p* value (padj = 9.73 × 10^−7^). Separate GO enrichment analyses showed that the downregulated genes produced a pattern broadly consistent with that of all candidate differentially expressed genes (Figure 7B). The downregulated genes were associated with 140 BP terms, of which four were significant, with defense response showing the smallest adjusted *p* value (padj = 2.81 × 10^−6^). They were also associated with 21 CC terms, including five significant terms, of which extracellular region was the most significant (padj = 3.30 × 10^−8^), and 99 MF terms, including 11 significant terms, of which signaling receptor binding was the most significant (padj = 3.20 × 10^−10^). In contrast, GO analysis of the upregulated genes identified 315 terms, none of which reached statistical significance (Figure 7C). These results suggest that the transcriptional changes associated with *LYPLA1* overexpression may predominantly involve extracellular signaling, cytokine-related functions, and heme or cofactor binding.

For KEGG analysis, 237 of the 548 candidate differentially expressed genes were successfully annotated to the KEGG database, and the corresponding effective background gene set comprised 7589 genes. Analysis of all candidate differentially expressed genes identified 265 KEGG pathways, 12 of which remained significantly enriched after multiple-testing correction (Figure 7D). These pathways included Steroid hormone biosynthesis, Cytokine–cytokine receptor interaction, Protein digestion and absorption, JAK–STAT signaling pathway, PI3K–Akt signaling pathway, Retinol metabolism, PPAR signaling pathway, TNF signaling pathway, Malaria, Butanoate metabolism, Rheumatoid arthritis, and Choline metabolism in cancer. Among these pathways, Steroid hormone biosynthesis showed the smallest adjusted *p* value (padj = 1.18 × 10^−4^) and contained 10 candidate differentially expressed genes, including seven upregulated and three downregulated genes. The PI3K–Akt signaling pathway was also significantly enriched (GeneRatio = 25/237, padj = 0.00604), involving 25 candidate differentially expressed genes, of which 11 were upregulated and 14 were downregulated. Among the 25 candidate differentially expressed genes associated with the PI3K–Akt signaling pathway, 19 remained significant after multiple-testing correction (adjusted *p* < 0.05), comprising 9 upregulated and 10 downregulated genes. Notably, *PDGFRA* was the most significantly upregulated gene, with an adjusted *p* value of 3.91 × 10^−21^. Given the role of PI3K–Akt signaling in cell proliferation and our previous findings that *LYPLA1* overexpression promoted cell proliferation, we hypothesized that this pathway may participate in *LYPLA1*-mediated regulation of cell proliferation. The PI3K–Akt signaling pathway was therefore selected for subsequent pharmacological intervention experiments. Separate KEGG analyses identified 224 pathways for the upregulated genes, six of which were significantly enriched (Figure 7E). The PPAR signaling pathway showed the smallest adjusted *p* value (padj = 1.19 × 10^−3^) and contained nine upregulated genes. The downregulated genes were associated with 159 KEGG pathways, of which 30 were significantly enriched (Figure 7F). Cytokine–cytokine receptor interaction was the most significantly enriched pathway (padj = 4.42 × 10^−8^) and contained 17 downregulated genes.

### 3.8. Determination of the Working Concentration of PI3K/AKT-IN-1

**Figure 8:**
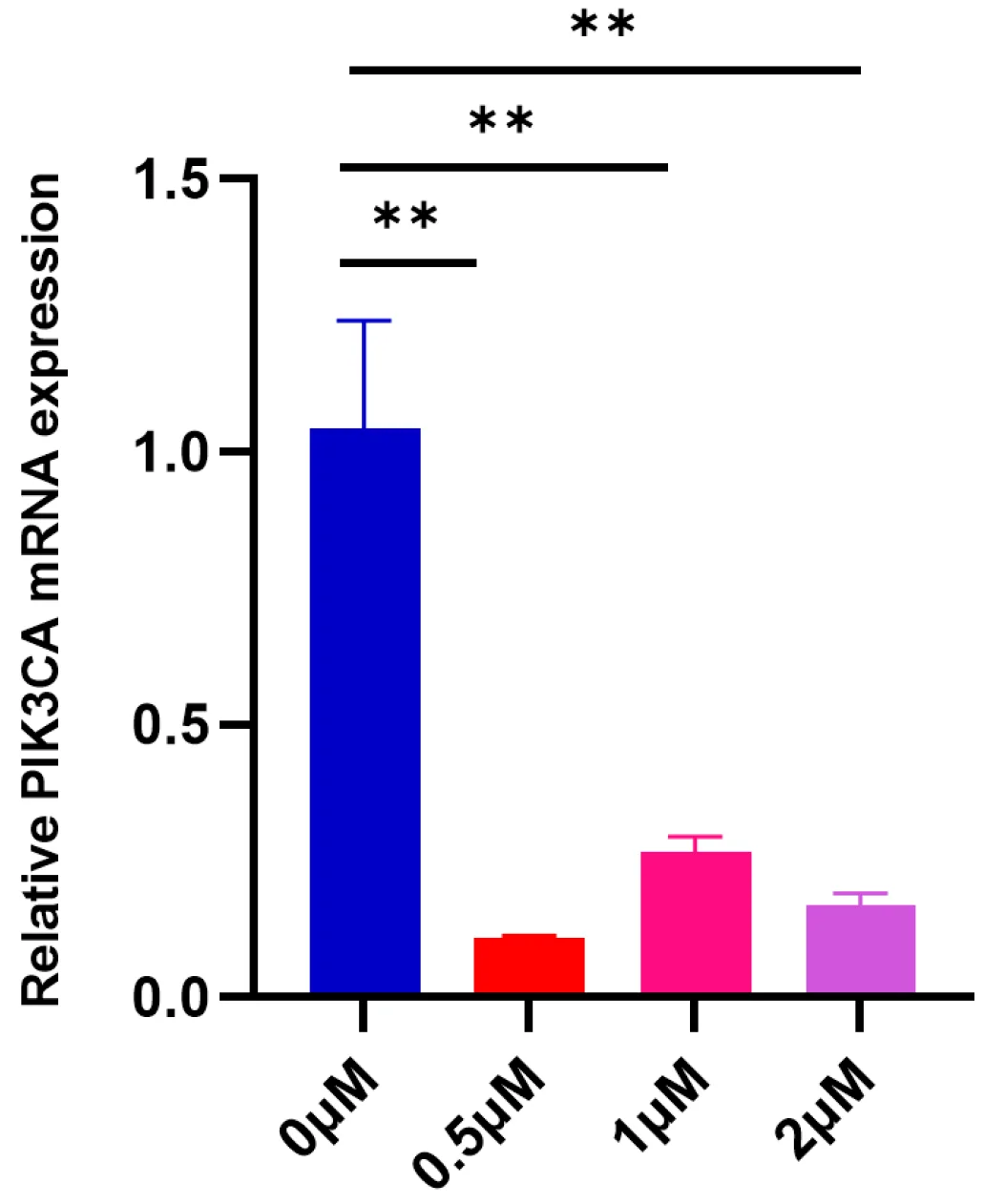
Relative expression levels of *PIK3CA* mRNA after treatment with different concentrations of the inhibitor Note: Statistical differences among groups were analyzed using one-way ANOVA followed by Dunnett’s multiple comparisons test. Data are presented as mean ± SEM, ** *p* < 0.01; n = 3.

To determine an appropriate working concentration of PI3K/AKT-IN-1 for subsequent experiments, cells were treated with 0, 0.5, 1, or 2 μM PI3K/AKT-IN-1, and *PIK3CA* mRNA expression was assessed by RT-qPCR. Compared with the 0 μM control group, *PIK3CA* mRNA expression was significantly reduced at all tested concentrations of PI3K/AKT-IN-1. Among the concentrations tested, the lowest *PIK3CA* mRNA expression level was observed in the 0.5 μM group (Figure 8). Increasing the concentration to 1 or 2 μM did not result in a further decrease in *PIK3CA* mRNA expression, indicating no apparent concentration-dependent reduction within the tested range. Therefore, 0.5 μM PI3K/AKT-IN-1 was selected as the working concentration for subsequent experiments based on the observed reduction in *PIK3CA* mRNA expression and the absence of a further decrease at higher concentrations.

### 3.9. Effects of PI3K/AKT-IN-1 on Cell Proliferation and CCND1 mRNA Expression

**Figure 9:**
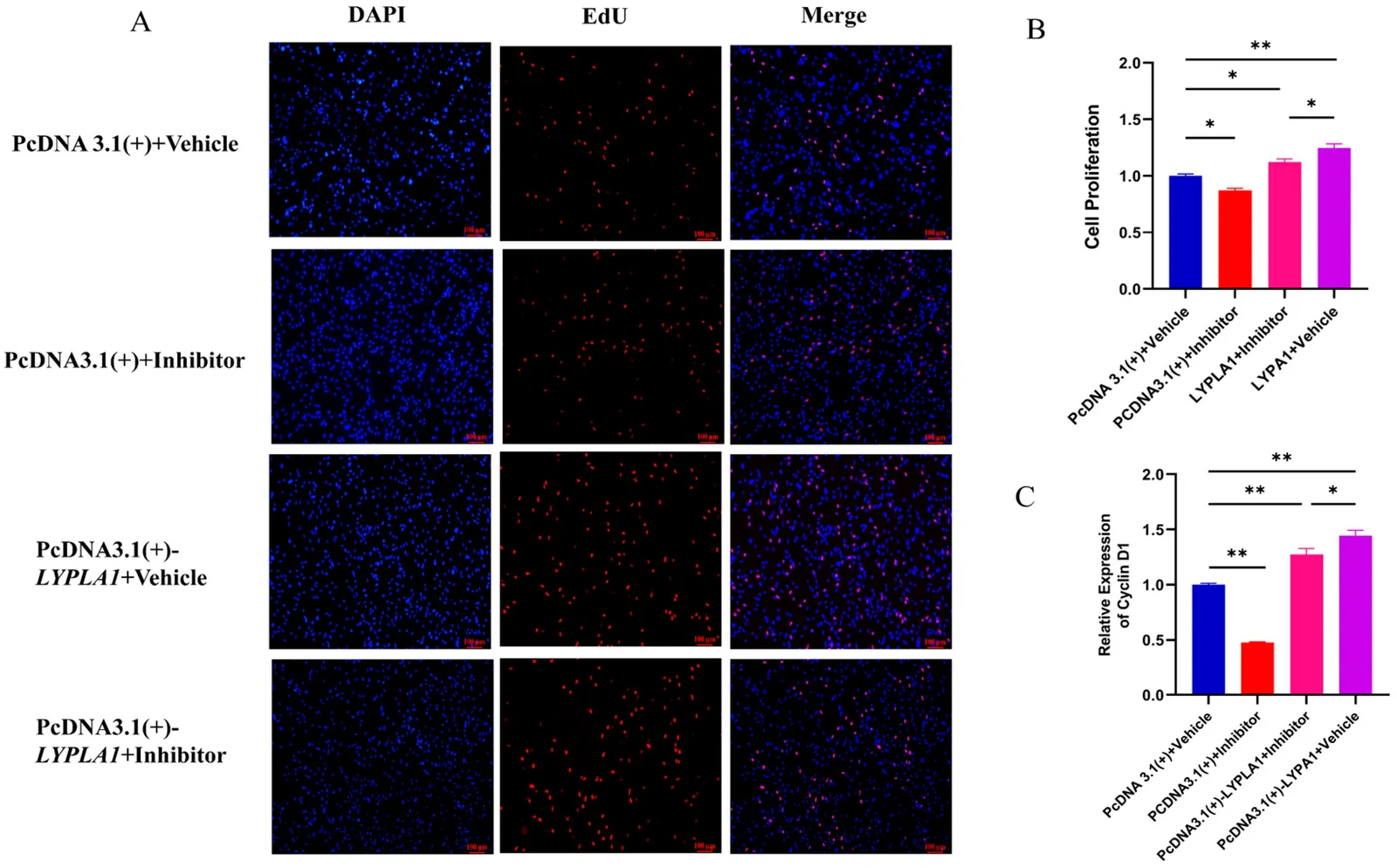
Effects of PI3K–Akt inhibition on *LYPLA1*-overexpression-associated cell proliferation and *CCND1* mRNA expression (**A**): Representative images of EdU staining in each group; blue indicates DAPI-stained cell nuclei, and red indicates EdU-positive cells; (**B**): Quantitative analysis of cell proliferation levels in each group; (**C**): Relative expression levels of *CCND1* mRNA in each group. Note: Statistical differences among groups were analyzed using one-way ANOVA followed by Holm–Šídák’s multiple comparisons test. Data are presented as mean ± SEM, n = 3; * *p* < 0.05, ** *p* < 0.01.

To further examine the potential involvement of PI3K–Akt signaling in *LYPLA1*-associated cell proliferation, a pharmacological inhibition experiment was performed using PI3K/AKT-IN-1. EdU staining showed that, compared with the empty vector plus vehicle group, PI3K/AKT-IN-1 treatment significantly decreased cell proliferation, whereas *LYPLA1* overexpression significantly increased cell proliferation. In *LYPLA1*-overexpressing cells, PI3K/AKT-IN-1 treatment significantly reduced cell proliferation compared with vehicle treatment, although the proliferation level remained significantly higher than that in the empty vector plus vehicle group (Figure 9A,B). RT-qPCR analysis showed a similar pattern. Compared with the empty vector plus vehicle group, PI3K/AKT-IN-1 treatment significantly reduced *CCND1* mRNA expression, whereas *LYPLA1* overexpression significantly increased *CCND1* mRNA expression. In *LYPLA1*-overexpressing cells, PI3K/AKT-IN-1 treatment significantly reduced *CCND1* mRNA expression compared with vehicle treatment, while *CCND1* expression remained significantly higher than that in the empty vector plus vehicle group (Figure 9C). Collectively, these findings suggest a potential association between *LYPLA1*-mediated cell proliferation and PI3K–Akt signaling. PI3K/AKT-IN-1 partially attenuated the *LYPLA1*-overexpression-associated increases in cell proliferation and *CCND1* mRNA expression. However, further studies, particularly analyses of PI3K–Akt pathway-related protein expression and phosphorylation, are required to confirm the involvement of this signaling pathway and clarify the underlying molecular mechanism.

## 4. Discussion

Previous research by our team found that *LYPLA1* overexpression promotes the proliferation and myogenic differentiation of fetal sheep myoblasts, whereas *LYPLA1* knockdown produces the opposite cellular phenotype [7]. Building on these findings, this study investigated the transcriptional changes resulting from *LYPLA1* overexpression and knockdown at the transcriptomic level and validated the transcriptomic results. Under strict FDR criteria, the number of differentially expressed genes between the two groups was not comparable: 362 significantly differentially expressed genes were identified in the overexpression group, whereas no FDR-significant differentially expressed genes were detected in the knockdown group. Therefore, this study concludes that *LYPLA1* overexpression is associated with a broad transcriptional response, while the results from the knockdown group are used solely for exploratory hypothesis generation. Pharmacological experiments showed that PI3K/AKT-IN-1 partially attenuated the proportion of EdU-positive cells and the elevated *Cyclin D1* expression associated with *LYPLA1* overexpression, suggesting that PI3K-Akt signaling may be involved in *LYPLA1*-related proliferative effects. *LYPLA1* overexpression was associated with alterations in the transcriptional state and proliferation-related phenotype of myoblasts in this single-donor culture system; however, its direct molecular targets and the causal relationship between *LYPLA1* and PI3K–Akt signaling remain to be elucidated.

APT1, encoded by *LYPLA1*, influences protein localization, stability, and signal transduction by regulating the S-palmitoylation status of proteins. Previous studies have shown that APT1 can remove palmitoyl groups from the G protein α-subunit and p21RAS [14], and inhibition of APT1 can also alter the subcellular localization and signal transduction of palmitoylated RAS [15]. Other studies have shown that APT1 can influence β-catenin stability and nuclear translocation, as well as downstream Cyclin D1 expression by regulating its palmitoylation [16]. These results suggest that the transcriptional changes induced by *LYPLA1* may be secondary effects resulting from alterations in the lipidation status of membrane signaling proteins or transcriptional co-regulators. Based on the findings of this study, a possible mechanism by which *LYPLA1* influences transcription is as follows: changes in the depalmitoylation status of proteins subsequently affect signal transduction or nuclear translocation, ultimately leading to a remodeling of downstream gene expression. Since this study has not yet examined the palmitoylation status or subcellular localization of specific substrates, this proposed mechanism remains to be verified.

The asymmetry in the results of overexpression and siRNA interference may be related to the intensity of the intervention and functional compensation among members of the *LYPLA* family. Wepy et al. found that when *LYPLA1* or *LYPLA2* was deleted individually, another hemolytic phospholipase could maintain cellular lipid homeostasis to some extent, whereas more pronounced accumulation of hemolytic phospholipids and cellular morphological changes only occurred upon simultaneous deletion of both [17], suggesting that *LYPLA2* or other depalmitoylases may partially buffer the effects of reduced *LYPLA1* levels. Although the candidate genes identified in the *LYPLA1* knockdown analysis did not pass FDR correction, *DNAH12*, *EXD1*, *FOXD4*, and *EYA2* showed directionally concordant expression changes across the two siRNA treatments targeting different *LYPLA1* sites. However, not all of these changes reached statistical significance in the siRNA-364 validation experiment. These findings therefore provide preliminary gene-level clues regarding potential transcriptional responses associated with reduced *LYPLA1* expression. *DNAH12* encodes the heavy chain of the axonemal motor protein and belongs to the microtubule-associated motor protein family [18]; its upregulation may reflect adaptive cellular regulation of microtubule dynamics, intracellular transport, or cytoskeletal homeostasis in the absence of *LYPLA1*. *EXD1* is involved in RNA binding, piRNA processing, and small RNA-mediated regulation [19]; its upregulation suggests that *LYPLA1* downregulation may be accompanied by changes in post-transcriptional regulation. *FOXD4* belongs to the forkhead box transcription factor family and is associated with developmental and cell fate-related transcriptional programs [20]; *EYA2*, in turn, acts in concert with *DACH2* and *SIX1* to participate in vertebrate muscle development and the regulation of myogenic genes [21]. Therefore, the directionally concordant changes in *FOXD4* and *EYA2* raise the hypothesis that reduced *LYPLA1* expression may be associated with alterations in developmental or myogenic transcriptional programs. The relationships described above represent associations between reduced *LYPLA1* expression and the responses of candidate genes; these associations require further experimental validation.

In the *LYPLA1* overexpression group, 362 differentially expressed genes (219 upregulated, 143 downregulated) were identified after FDR adjustment; *GPM6B*, *PRSS35*, and *ACTC1* showed an upregulated trend, while *MYO1G*, *CSF2*, and *CSF3* were downregulated. *GPM6B* encodes the membrane glycoprotein M6B, which is associated with membrane-related functions and cell differentiation [22]; given that LYPLA1/APT1 regulates protein palmitoylation and membrane localization, the upregulation of *GPM6B* may be a secondary marker of altered membrane structure or signaling status, rather than a confirmed direct substrate. *PRSS35* is associated with the composition of the extracellular matrix and collagen-related proteins [23]; its upregulation correlates with the enrichment of the extracellular domain, receptor binding, and intercellular communication categories in this study, suggesting that high *LYPLA1* expression may be accompanied by remodeling of the cell–matrix microenvironment. *ACTC1* encodes alpha-cardiac actin, a structural component of the contractile cytoskeleton and its upregulation can be regarded as a candidate indicator of enhanced myogenic structures and cytoskeletal programs [24]; *MYO1G* is involved in the coupling of the plasma membrane to the actin cytoskeleton, membrane dynamics, and cell migration [25]; its downregulation suggests that *LYPLA1* overexpression may alter the membrane–cytoskeleton coupling state.

The enrichment of entries related to defense responses, immune system processes, the extracellular space, and cytokines in the *LYPLA1* overexpression group suggests that elevated *LYPLA1* levels may be accompanied by alterations in transcriptional programs associated with extracellular signaling and intercellular communication. Previous studies have shown that myogenic cytokines can participate in myoblast differentiation and skeletal muscle regeneration through autocrine mechanisms [26]. In particular, the downregulation of *CSF2* and *CSF3* provides specific transcript-level evidence for these enrichment findings and is consistent with the changes in cytokine–cytokine receptor interactions observed in the overexpression group. Combined with the results of the PI3K-Akt pharmacological intervention in this study, changes in extracellular signaling and receptor-related pathways may be associated with the regulation of downstream proliferative signals; however, mRNA changes alone are insufficient to determine the secretion levels of the corresponding proteins or their net effects on proliferation or differentiation. Subsequent measurement of the levels of *CSF2*, *CSF3*, and other representative cytokines in culture supernatants could further determine whether these transcriptional changes translate into secretory phenotypes.

The enrichment of genes associated with PPAR signaling and steroid hormone biosynthesis further suggests a potential link between *LYPLA1* and cellular lipid homeostasis. A recent study in Erhualian pigs demonstrated that *LYPLA1* promotes intramuscular preadipocyte differentiation and lipid deposition by modulating phosphatidylcholine levels [27]. This finding is broadly consistent with the enrichment of lipid metabolism-related pathways observed in the present study. However, the two cell types have distinct functional contexts: preadipocytes are primarily characterized by lipid storage and lipid droplet formation, whereas the present study used fetal ovine myoblasts maintained under growth conditions. Therefore, changes in PPAR-related genes in myoblasts should not be interpreted as direct evidence of enhanced adipogenesis; rather, they may reflect adaptive alterations in membrane phospholipid composition, fatty acid utilization, or energy metabolism. Because phosphatidylcholine and triglyceride levels, as well as PPAR transcriptional activity, were not assessed in the present study, the current findings can only be interpreted as indicating an association between *LYPLA1* overexpression and alterations in the lipid metabolism-related transcriptional network. The specific metabolic consequences require further investigation.

The PI3K–Akt signaling pathway links growth factor stimulation to cellular metabolism and cell-cycle regulation. In primary bovine myoblasts, *PLAG1* promotes cell proliferation and inhibits apoptosis, accompanied by changes in the phosphorylation levels of PI3K and AKT and in the abundance of cell-cycle-related proteins such as Cyclin D1. Pathway inhibition and activation experiments further supported the involvement of PI3K–Akt signaling in these biological effects [28]. In the present study, PI3K/AKT-IN-1 partially reduced the increases in the proportion of EdU-positive cells and *CCND1* mRNA expression associated with *LYPLA1* overexpression, suggesting that PI3K–Akt signaling contributes to the proliferative effects associated with *LYPLA1*. However, these parameters were not fully restored to control levels following inhibitor treatment, indicating that the effects of *LYPLA1* may also involve other signaling pathways or metabolic processes. In addition, pathway activity markers such as p-AKT, p-GSK3β, and p-mTOR were not examined in this study. Therefore, the current findings support the involvement of PI3K–Akt signaling in the observed proliferative phenotype but are insufficient to demonstrate that *LYPLA1* directly activates this pathway.

In summary, this study expands our understanding of the role of *LYPLA1* in fetal ovine myoblasts at both the transcriptomic and pharmacological levels. In the *LYPLA1* knockdown group, the upregulation of *DNAH12* and *EXD1*, together with the downregulation of *FOXD4* and *EYA2*, provides exploratory evidence that reduced *LYPLA1* expression may be accompanied by alterations in microtubule-dependent transport, post-transcriptional regulation, and myogenic transcriptional networks. In the overexpression group, the upregulation of *GPM6B, PRSS35*, and *ACTC1*, along with the downregulation of *MYO1G*, *CSF2*, and *CSF3*, links *LYPLA1* to membrane–cytoskeletal coupling, the extracellular microenvironment, myogenic structural organization, and cytokine regulation. The partial antagonistic effect of PI3K/AKT-IN-1 suggests that PI3K–Akt signaling may contribute to the proliferative effects associated with *LYPLA1*. *LYPLA1* may indirectly influence cellular signaling and gene expression by regulating protein depalmitoylation. However, whether the identified candidate genes represent key downstream mediators of *LYPLA1* remains to be established. Because all cultures were derived from a single fetal donor, the culture replicates do not capture between-animal biological variability, which limits the generalizability of the present findings. Future studies using independent fetal donors, together with genetic rescue experiments, functional perturbation of candidate genes, and analyses of protein phosphorylation and lipidation, will be required to validate these findings and clarify the downstream mechanisms of *LYPLA1*.

## 5. Conclusions

*LYPLA1* appears to be a potential modulator of the transcriptional state and proliferative behavior of fetal ovine myoblasts, with the most pronounced response observed following *LYPLA1* overexpression. The transcriptomic and pharmacological findings together suggest that PI3K-Akt signaling contributes to, but is unlikely to fully account for, the proliferative phenotype associated with elevated *LYPLA1* expression. These results identify candidate genes and pathways for further investigation of skeletal muscle growth and may contribute to a better understanding of the genetic regulation of muscle development in sheep. These findings should be interpreted cautiously because all cultures were derived from a single fetal donor, pathway activation was not confirmed at the protein phosphorylation level, and LYPLA1 protein abundance and depalmitoylation activity were not directly assessed. Future studies using independent animal donors, genetic rescue or pathway-manipulation approaches, and direct analyses of AKT phosphorylation and *LYPLA1*-dependent protein lipidation are warranted to further establish causality and clarify the underlying mechanisms.

## Figures and Tables

**Table 1:** Sequence information for siRNA and Negative Control (NC).

Name	Primer Name	Sequence (5′-3′)
*LYPLA1*	siRNA-364	F:GGAGCUUUAUCUCUGUACATT
		R:UGUACAGAGAUAAAGCUCCTT
	siRNA-208	F:CCAUCAUGGUUUGACAUUATT
		R:UAAUGUCAAACCAUGAUGGTT
	siRNA-1947	F:AUUUUUCAAACUAUCGGUUTT
		R:AACCGAUAGUUUGAAAAAUTT
NC	NC-F	UUCUCCGAACGUGUCACGUTT
	NC-R	ACGUGACACGUUCGGAGAATT

**Table 2:** Primers used in plasmid construction.

Name	Primer Name	Sequence (5′-3′)	Product Length
*LYPLA1*	*LYPLA1*-F	gggagacccaagctggctagcTGTATGTGCGGCAATAACATGTC	679 bp
	*LYPLA1*-R	aacgggccctctagactcgagGACGATGCTGGTGTGCACTTC	

Note: In the primer sequences, the lowercase letters at the 5′ end represent the 15-bp regions homologous to the termini of the linearized pcDNA3.1(+) vector, the underlined lowercase letters indicate the restriction sites, and the uppercase letters correspond to the inserted fragment.

**Table 3:** Cell Transfection System.

Culture Vessel	Group	Amount of siRNA (pmol) 50 nM	Amount of DNA (μg)	Volume of jetPRIME Reagent (μL)	Volume of jetPRIME Buffer for Complex Formation (μL)	Volume of Growth Medium (mL)
6-well	NC	110	0	4	200	2
	siRNA-364	110	0	4	200	2
	siRNA-Mock	0	0	4	200	2
	pcDNA 3.1(+)	0	2	4	200	2
	pcDNA 3.1(+)-*LYPLA1*	0	2	4	200	2
	Plasmid-Mock	0	0	4	200	2

Note: The final concentration of siRNA for transfection was 50 nM; the mock group received only jetPRIME transfection reagent and buffer, with no siRNA or plasmid DNA added.

**Table 4:** Gene-Specific Primers Used for RT-qPCR.

Gene Name	Primer Name	Sequence (5′-3′)	GenBank Accession
*LYPLA1*	*LYPLA1*-F	GATTGGGAGACACAGGGCAT	NM_001306103.1
	*LYPLA1*-R	GCATGCGGGCAGATGTATTT	
*DNAH12*	*DNAH12*-F	GCAGGTGCAAGCAAGGAATTTT	XM_012099851.5
	*DNAH12*-R	GGTTTGATGAACGGTGAGCC	
*EXD1*	*EXD1*-F	AGTTTGGTGCTGCAATGCTC	XM_060419061.1
	*EXD1*-R	GTCCATTGTTGAAAGCGCGA	
*FOXD4*	*FOXD4*-F	ATCGTGTTCCTTGCGGGTTT	XM_060409399.1
	*FOXD4*-R	CGTCTCTGTGTGATCCCTCC	
*EYA2*	*EYA2*-F	AGTGGCGGCAGCAGAG	XM_027977274.2
	*EYA2*-R	CCGATGTGGTGATGCCTTCT	
*PRSS35*	*PRSS35*-F	GGAAGCTTCGCAGGACAAGA	XM_004011131.5
	*PRSS35*-R	GCATTCGATGCCACACACTC	
*ACTC1*	*ACTC1*-F	CTGGATCTAGCTGGTCGGGA	XM_004010972.6
	*ACTC1*-R	ATGCCAGCTGATTCCATACCAAT	
*TMEM40*	*TMEM40*-F	CAGAGAGTCTCAGGCATTCCA	XM_027957796.2
	*TMEM40*-R	GATGTCCTGAGGGTTGGGTG	
*MYO1G*	*MYO1G*-F	CAGCCACGTCAGAAAGGTGA	XM_027968703.2
	*MYO1G*-R	CAGCTTGTAGAGGTGCCGAT	
*GAPDH*	*GAPDH*-F	TCTCAAGGGCATTCTAGGCTAC	NM_001190390.1
	*GAPDH*-R	GCCGAATTCATTGTCGTACCAG	

**Table 5:** Specific primers used for RT-qPCR.

Gene Name	Primer Name	Sequence (5′–3′)	GenBank Accession
*cyclin D1*	*cyclin D1*-F	GCTTCCTCTCCTATCACCGC	XM_027959928.2
	*cyclin D1*-R	GGCTTTGGGGTCCAAGTTCT	
*PIK3CA*	*PIK3CA*-F	GGAGCCCCGAGCATTTCTG	XM_015092541.3
	*PIK3CA*-R	AGGATTCTTGGGGGCATCAA	

**Table 6:** Quality Statistics for Transcriptome Sequencing Data from Each Sample.

Groups	Samples	Raw Reads	Clean Reads	Q20/%	Q30/%	GC Content/%
PCDNA3.1(+)	PC1	56162380	53500338	99.16	96.29	55.9
	PC2	50408742	48775866	99.17	96.41	54.66
	PC3	48640826	47093384	99.15	96.35	54.73
PCDNA3.1(+)-*LYPLA1*	L1	50583122	49231012	99.21	96.52	53.85
	L2	46759774	45513498	99.17	96.4	54.05
	L3	50444920	49320170	99.19	96.48	53.97
NC	NC1	47912690	46423406	99.23	96.57	53.28
	NC2	53377870	51732774	99.21	96.47	53.25
	NC3	54077538	52722252	99.22	96.52	52.42
siRNA-364	siRNA364-1	51223822	49825370	99.2	96.51	52.51
	siRNA364-2	48932198	47404848	99.24	96.61	51.48
	siRNA364-3	47267370	45676198	99.22	96.56	52.91

**Table 7:** Top Five Nominally Significant Candidate Genes Upregulated in the siRNA-364 Group Compared to the NC Group.

Gene id	log2FoldChange	*p* Value	Gene Name	Gene Chr
101108960	5.11 × 10^0^	1.08 × 10^−3^	*DNAH12*	NC-056072.1
101123417	4.55 × 10^0^	3.53 × 10^−2^	*LOC101123417*	NC-056073.1
132658134	4.40 × 10^0^	1.95 × 10^−2^	*LOC132658134*	NC-056054.1
101115129	4.23 × 10^0^	2.88 × 10^−2^	*EXD1*	NC-056060.1
114113677	4.11 × 10^0^	3.23 × 10^−2^	*LOC114113677*	NC-056056.1

**Table 8:** Top Five Nominally Significant Candidate Genes Downregulated in the siRNA-364 Group Compared to the NC Group.

Gene id	log2FoldChange	*p* Value	Gene Name	Gene Chr
101116383	−4.24 × 10^0^	1.74 × 10^−2^	*FOXD4*	NC-056055.1
101118806	−4.22 × 10^0^	3.27 × 10^−2^	*EYA2*	NC-056066.1
121817249	−4.15 × 10^0^	2.97 × 10^−2^	*LOC121817249*	NC-056072.1
101112183	−4.10 × 10^0^	4.03 × 10^−2^	*GNG13*	NC-056077.1
114114341	−4.09 × 10^0^	2.84 × 10^−2^	*TRNAI-UAU*	NC-056056.1

**Table 9:** Top Five Genes Upregulated in PC-*LYPLA1* Compared to PC.

Gene id	log2FoldChange	*p* Value	Padj	Gene Name	Gene-Chr
101107971	4.11516866754704	1.00 × 10^−156^	1.41 × 10^−152^	*LYPLA1*	NC-056062.1
101120549	3.37090948913995	1.49 × 10^−6^	2.07 × 10^−5^	*GPM6B*	NC-056080.1
114115289	3.19188070702576	3.39 × 10^−100^	2.38 × 10^−96^	*LOC114115289*	NC-056059.1
101115799	3.08443776423369	4.31 × 10^−4^	2.44 × 10^−3^	*PRSS35*	NC-056061.1
101111554	2.59842084086919	1.53 × 10^−8^	4.00 × 10^−7^	*ACTC1*	NC-056060.1

Note: The genes listed in the table are differentially expressed genes identified by comparing the PC-*LYPLA1* group with the PC group. The selection criteria were padj ≤ 0.05 and |log2FoldChange| ≥ 1, and the top five genes were selected based on their log2FoldChange values.

**Table 10:** Top Five Genes Downregulated in PC-*LYPLA1* Compared to PC.

Gene id	log2FoldChange	*p* Value	Padj	Gene Name	Gene-Chr
101102938	−3.281733242	2.50 × 10^−5^	2.23 × 10^−4^	*TMEM40*	NC-056072.1
101105265	−2.627237204	1.73 × 10^−3^	7.67 × 10^−3^	*LOC101105265*	NC-056064.1
101114605	−2.595194502	8.24 × 10^−5^	6.05 × 10^−4^	*MYO1G*	NC-056057.1
443523	−2.562093632	4.88 × 10^−12^	3.08 × 10^−10^	*CSF3*	NC-056064.1
443400	−2.316230574	5.91 × 10^−14^	6.39 × 10^−12^	*CSF2*	NC-056058.1

Note: The genes listed in the table are differentially expressed genes identified by comparing the PC-*LYPLA1* group with the PC group. The selection criteria were padj ≤ 0.05 and |log2FoldChange| ≥ 1, and the top five genes were selected based on their log2FoldChange values.

## Data Availability

The original contributions presented in this study are included in the article/Supplementary Material. The raw RNA-sequencing data generated in this study have been deposited in the NCBI Sequence Read Archive under BioProject accession PRJNA1504272.

## Supplementary Materials

The following supporting information can be downloaded at https://dataview.ncbi.nlm.nih.gov/object/PRJNA1504272?reviewer=l1ud4mrgqn9fc82sc5efl8p6qu (accessed on 16 August 2026). The raw data have been uploaded to the NCBI. The complete list of differentially expressed genes (DEGs), together with the full results of the GO and KEGG enrichment analyses—including enrichment results for all DEGs, upregulated genes, and downregulated genes—as well as the exon/intron/intergenic read distribution results, raw-count matrix, and relevant analysis parameters, are provided in the Supplementary Files to facilitate transparency and reproducibility.
